# Deep brain stimulation of the hypothalamic region: a systematic review

**DOI:** 10.1007/s00701-025-06430-w

**Published:** 2025-02-04

**Authors:** Mohammad Mofatteh, Abdulkadir Mohamed, Mohammad Sadegh Mashayekhi, Georgios P. Skandalakis, Clemens Neudorfer, Saman Arfaie, ArunSundar MohanaSundaram, Mohammadmahdi Sabahi, Ayush Anand, Rabii Aboulhosn, Xuxing Liao, Andreas Horn, Keyoumars Ashkan

**Affiliations:** 1 School of Medicine, Dentistry and Biomedical Sciences, Queen’s University Belfast, 97 Lisburn Road, Belfast, BT9 7BL UK; 2 Neuro International Collaboration (NIC), London, UK; 3 Medical Sciences Division, University of Oxford, Oxford, UK; 4 Neuro International Collaboration (NIC), Oxford, UK; 5 Faculty of Medicine, Division of Neurosurgery, University of Ottawa, Ottawa, ON Canada; 6 Neuro International Collaboration (NIC), Vancouver, Ottawa, ON Canada; 7 Department of Neurosurgery, Evangelismos General Hospital, National and Kapodistrian University of Athens, Athens, Greece; 8 Section of Neurosurgery, Dartmouth Hitchcock Medical Center, Lebanon, NH USA; 9 Brain Modulation Lab, Department of Neurosurgery, Massachusetts General Hospital, Boston, MA USA; 10 Harvard Medical School, Boston, MA USA; 11 Center for Brain Circuit Therapeutics Department of Neurology Brigham & Women’s Hospital, Harvard Medical School, Boston, MA USA; 12 Movement Disorder and Neuromodulation Unit, Department of Neurology, Charité -Universitätsmedizin Berlin, corporate member of, Freie Universität Berlin and Humboldt-Universität Zu Berlin, Berlin, Germany; 13 Department of Neurology and Neurosurgery, McGill University, Montreal, QC Canada; 14 Division of Neurosurgery, Department of Clinical Neurological Sciences, Schulich School of Medicine and Dentistry, University of Western Ontario, London, ON Canada; 15 Department of Molecular and Cell Biology, University of California Berkeley, Berkeley, CA USA; 16 Neuro International Collaboration (NIC), Montreal, QC Canada; 17 School of Pharmacy, Sathyabama Institute of Science and Technology, Chennai, Tamilnadu India; 18 Department of Neurological Surgery, Pauline Braathen Neurological Center, Cleveland Clinic Florida, Weston, FL USA; 19 Koirala Institute of Health Sciences, B. P, Dharan, Nepal; 20 School of Medicine, University of Aberdeen, Aberdeen, UK; 21 Department of Neurosurgery, First People’s Hospital of Foshan, Foshan, Guangdong Province China; 22 Movement Disorder and Neuromodulation Unit, Department of Neurology, Charité - Universitätsmedizin Berlin, Berlin, Germany; 23 Center for Brain Circuit Therapeutics, Department of Neurology, Brigham and Women’s Hospital, Boston, MA USA; 24 Departments of Neurology and Neurosurgery, Massachusetts General Hospital, Boston, MA USA; 25 School of Biomedical Engineering and Imaging Sciences, Faculty of Life Sciences and Medicine, King’s College London, London, UK; 26 King’s Health Partners Academic Health Sciences Centre, London, UK; 27 Department of Basic and Clinical Neuroscience, Institute of Psychiatry, Psychology and Neuroscience, King’s College London, London, UK; 28 Department of Neurosurgery, King’s College Hospital NHS Foundation Trust, London, UK

**Keywords:** Deep brain stimulation, DBS, Hypothalamus, Hypothalamic nuclei, Aggression, Refractory headaches

## Abstract

**Background:**

Deep brain stimulation (DBS) has been successfully used for the treatment of circuitopathies including movement, anxiety, and behavioral disorders. The hypothalamus is a crucial integration center for many peripheral and central pathways relating to cardiovascular, metabolic, and behavioral functions and constitutes a potential target for neuromodulation in treatment-refractory conditions. To conduct a systematic review, investigating hypothalamic targets in DBS, their indications, and the primary clinical findings.

**Methods:**

PubMed, Scopus, and Web of Science databases were searched in accordance with the PRISMA guideline to identify papers published in English studying DBS of the hypothalamus in humans.

**Results:**

After screening 3,148 papers, 34 studies consisting of 412 patients published over two decades were included in the final review. Hypothalamic DBS was indicated in refractory headaches (*n* = 238, 57.8%), aggressive behavior (*n* = 100, 24.3%), mild Alzheimer’s disease (*n* = 58, 14.1%), trigeminal neuralgia in multiple sclerosis (*n* = 5, 1.2%), Prader-Willi syndrome (*n* = 4, 0.97%), and atypical facial pain (*n* = 3, 0.73%). The posterior hypothalamus was the most common DBS target site across 30 studies (88.2%). 262 (63.6%) participants were males, and 110 (26.7%) were females. 303 (73.5%) patients were adults whereas 33 (8.0%) were pediatrics. The lowest mean age of participants was 15.25 ± 4.6 years for chronic refractory aggressiveness, and the highest was 68.5 ± 7.9 years in Alzheimer’s disease patients. The mean duration of the disease ranged from 2.2 ± 1.7 (mild Alzheimer’s disease) to 19.8 ± 10.1 years (refractory headaches). 213 (51.7%) patients across 29 studies (85.3%) reported symptom improvements which ranged from 23.1% to 100%. 25 (73.5%) studies reported complications, most of which were associated with higher voltage stimulations.

**Conclusions:**

DBS of the hypothalamus is feasible in selected patients with various refractory conditions ranging from headaches to aggression in both pediatric and adult populations. Future large-scale studies with long-term follow-up are required to validate the safety and efficacy data and extend these findings.

**Supplementary Information:**

The online version contains supplementary material available at 10.1007/s00701-025-06430-w.

## Introduction

With a volume of 4 cm^3^, occupying 0.3% of the adult human brain, the hypothalamus is an intricate neuroanatomical region responsible for neuroendocrine, behavioral, and autonomic processes critical to life [7, 22, 49, 71]. As a highly connected neural structure, the hypothalamus is at the crossroads of receiving signals from corticolimbic structures, brainstem nuclei, and the spinal cord [40, 61]. Hypothalamic nuclei integrate the information received, and alter it within secondary and tertiary hypothalamic structures, before relaying it to autonomic and limbic control centers where appropriate physiological responses are elicited [7, 71, 72]. Additionally, the hypothalamus communicates with the pituitary gland to orchestrate physiological responses by releasing various hormones and factors via the hypothalamic–pituitary–adrenal axis (HPA) [43, 78].

Since the introduction of the first hypothalamic DBS application by Sano and colleagues in 1970 to treat 51 patients with pathologically aggressive behavior, which resulted in reduction of aggression in 95% of cases [69, 70], stereotactic approaches targeting the hypothalamus have opened possibilities for treating various psychological, behavioral, and neurological conditions [4, 12, 36, 39, 44, 54, 64, 67, 79, 81]. This article reviews the functional organization of the hypothalamus, the effects of deep brain stimulation (DBS) on different hypothalamic regions, and the stereotactic targets that have most often been used in the treatment of psychopathological and behavioral symptoms. To the best of our knowledge, this is the first systematic review with a focus on DBS of the hypothalamus.

## Methods

### Search strategy

Three databases of PubMed, Scopus, and Web of Science were searched from the inception to May 25, 2023, following the Preferred Reporting Items for Systematic Reviews and Meta-Analyses (PRISMA) guidelines [60]. Details of search terms used for each database are shown in Supplementary Table 1. The study was not registered in PROSPERO registry.

### Inclusion and exclusion criteria

Eligibility criteria were 1) original articles, 2) focused on DBS of the hypothalamus and associated areas only as clearly reported, 3) included human subjects only, and 4) had at least four subjects. Exclusion criteria were 1) articles not in English, 2) case reports with fewer than four subjects, and 3) articles where meaningful data extraction was not possible.

### Data extraction

Extracted data are presented in Tables 1–7. Microsoft Excel (version 2016; Microsoft) was used for all calculations.

**Table 1:** An overview of studies included in the final review

Study	Title	Journal	Country	Single/ Multi-center	Objective
Leone et al., 2003 [45]	Hypothalamic deep brain stimulation for intractable chronic cluster headache: A 3-year follow-up	Neurological Sciences	Italy	Single-center	To test whether DBS of ipsilateral posterior inferior hypothalamus gives an improvement in the therapy of refractory CCH patients
Franzini et al., 2004 [29]	Hypothalamic deep brain stimulation for the treatment of chronic cluster headaches: A series report	Neuromodulation	Italy	Single-center	To explore the effect of chronic high-frequency stimulation of PH for the treatment of CCH
Schoenen, et al., 2005 [73]	Hypothalamic stimulation in chronic cluster headache: A pilot study of efficacy and mode of action	Brain	Belgium	Single-center	To test if refractory CCH patients can be helped with ipsilateral ventroposterior hypothalamus DBS using previously published stereotactic coordinates along with investigating potential mechanisms of action
Leone et al., 2006a [46]	Acute hypothalamic stimulation and ongoing cluster headache attacks	Neurology	Italy	Single-center	To examine the effect of acute hypothalamic stimulation on CH attacks in patients implanted to prevent CCH
Leone et al., 2006b [47]	Hypothalamic stimulation for intractable cluster headache: Long-term experience	Neurology	Italy	Single-center	To report on the long-term outcomes of continuous hypothalamic stimulation in patients with refractory CH
May et al., 2006 [55]	Hypothalamic deep brain stimulation in positron emission tomography	The Journal of Neuroscience	Italy	Single-center	To find the mechanism for DBS in CCH in operated patients, using positron emission tomography and switching the hypothalamic stimulator on and off
Broggi et al., 2007 [14]	Update on neurosurgical treatment of chronic trigeminal autonomic cephalalgias and atypical facial pain with deep brain stimulation of posterior hypothalamus: Results and comments	Neurological Sciences	Italy	Single-center	To describe the effect and problems of DBS of the PH over 7 years, for treatment of chronic trigeminal autonomic cephalalgias and atypical facial pain
Cortelli et al., 2007 [20]	Effect of deep brain stimulation of the posterior hypothalamic area on the cardiovascular system in chronic cluster headache patients	European Journal of Neurology	Italy	Single-center	To determine the cardiovascular effects of chronic stimulation of the PH in CH patients
Starr et al., 2007 [82]	Chronic stimulation of the posterior hypothalamic region for cluster headache: Technique and 1-year results in four patients	Journal of Neurosurgery	US	Single-center	To report approach, intraoperative observations, and 1-year results after hypothalamic DBS in patients with refractory CH
Bartsch et al., 2008 [8]	Hypothalamic deep brain stimulation for cluster headache: Experience from a new multicase series	Cephalalgia	Germany	Multi-center	To report the results of patients with CCH in whom a DBS in the PH was done
Cordella et al., 2009 [19]	Hypothalamic stimulation for trigeminal neuralgia in multiple sclerosis patients: Efficacy on the paroxysmal ophthalmic pain	Multiple Sclerosis	Italy	Single-center	To assess the role of PH DBS in the treatment of trigeminal neuralgia in multiple sclerosis patients
Jurgens et al., 2009 [37]	Hypothalamic deep-brain stimulation modulates thermal sensitivity and pain thresholds in cluster headache	Pain	Germany and Italy	Multi-center	To investigate whether hypothalamic DBS affects thermal and pain perception only in the clinically affected region or in other regions
Fontaine et al., 2010a [25]	Anatomical location of effective deep brain stimulation electrodes in chronic cluster headache	Brain	France	Multi-center	To identify the structures activated and involved in the therapeutic effect, using stereotactic localization of the stimulating contacts projected on stereotactic anatomic atlases
Fontaine et al., 2010b [26]	Safety and efficacy of deep brain stimulation in refractory cluster headache: A randomized placebo-controlled double-blind trial followed by a 1-year open extension	The Journal of Headache and Pain	France	Multi-center	To assess the efficacy and safety of unilateral hypothalamic DBS in severe refractory CCH
Sillay et al., 2010 [77]	Deep brain stimulation for medically intractable cluster headache	Neurobiology of Disease	US	Single-center	To review features of CH, surgical indications for DBS, surgical techniques, clinical outcomes, and possible mechanisms
Seijo et al., 2011 [74]	Neuromodulation of the posterolateral hypothalamus for the treatment of chronic refractory cluster headache: Experience in five patients with a modified anatomical target	Cephalalgia	Spain	Single-center	To report long-term outcomes of patients with refractory CCH with DBS of a modified target
Franzini et al., 2013 [28]	Deep-brain stimulation for aggressive and disruptive behavior	World Neurosurgery	Italy	Single-center	To describe experience with DBS used in the treatment of refractory aggressive and disruptive behavior
Leone et al., 2013 [48]	Success, failure, and putative mechanisms in hypothalamic stimulation for drug-resistant chronic cluster headache	Pain	Italy	Single-center	To report long-term follow-up of hypothalamic stimulation for refractory CCH
Torres et al., 2013 [87]	Long-term results of posteromedial hypothalamic deep brain stimulation for patients with resistant aggressiveness	Journal of Neurosurgery	Spain	Single-center	To examine the long-term outcome of PMH DBS in patients with severe erethism
Piacentino et al., 2014 [66]	Drug-resistant cluster headache: long-term evaluation of pain control by posterior hypothalamic deep-brain stimulation	World Neurosurgery	Italy	Single-center	To review the outcome of patients affected by refractory CH treated by PH DBS with follow-up for more than 5 years
Benedetti-Isaac et al., 2015 [10]	Seizure frequency reduction after posteromedial hypothalamus deep brain stimulation in drug-resistant epilepsy associated with intractable aggressive behavior	Epilepsia	Colombia	Single-center	To analyze the result of DBS of the PMH on seizure frequency in patients with refractory epilepsy associated with refractory aggressive behavior
Akram et al., 2017 [2]	Optimal deep brain stimulation site and target connectivity for chronic cluster headache	Neurology	UK	Single-center	To investigate the mechanism of DBS for refractory CCH and the optimal target in the ventral tegmental area
Micieli et al., 2017 [56]	Single-unit analysis of the human posterior hypothalamus and red nucleus during deep brain stimulation for aggressivity	Journal of Neurosurgery	Colombia	Single-center	To compare the properties of single-unit recordings in the human PH, ventral thalamus, and red nucleus and report on the effectiveness of bilateral PH DBS as a treatment for aggressive behavior in patients
Seijo-Fernandez et al., 2018 [75]	Long-Term results of deep brain stimulation of the mamillotegmental fasciculus in chronic cluster headache	Stereotactic and Functional Neurosurgery	Spain	Single-center	To describe the results of patients with CCH with DBS with 5 years mean follow-up, and to identify the anatomic structures stimulated by DBS
Franco et al., 2018 [27]	Assessment of safety and outcome of lateral hypothalamic deep brain stimulation for obesity in a small series of patients with Prader-Willi syndrome	JAMA Network Open	Brazil	Single-center	To test the safety and analyze the outcome of DBS in patients with Prader-Willi syndrome
Nowacki et al., 2019 [63]	Deep brain stimulation of chronic cluster headaches: Posterior hypothalamus, ventral tegmentum and beyond	Cephalalgia	UK	Single-center	To provide long-term follow-up results and analysis of stimulation sites of patients with CCH undergoing DBS of the ipsilateral PH
Belvis et al., 2020 [9]	Efficacy and safety of surgical treatment of cluster headache	Medicina Clinica	Spain	Single-center	To report 15 years of experience of procedures performed in consecutive patients with refractory CCH
Benedetti-Isaac et al., 2021 [11]	Deep brain stimulation in the posteromedial hypothalamic nuclei in refractory aggressiveness: Post-surgical results of 19 cases	International Journal of Neuropsychopharmacology	Colombia	NS	To provide long-term follow-up results on the efficacy of PMH DBS for refractory aggression
Contreras Lopez et al., 2021 [18]	Directional deep brain stimulation of the posteromedial hypothalamus for refractory intermittent explosive disorder: A case series using a novel neurostimulation device and intraoperative microdialysis	World Neurosurgery	Colombia	Single-center	To test the efficacy and safety of the PMH DBS using 8-contact directional leads in patients with the refractory explosive disorder
Neudorfer et al., 2021 [62]	Mapping autonomic, mood and cognitive effects of hypothalamic region deep brain stimulation	Brain	Canada	Multi-center	To explore the acute sequelae of hypothalamic region DBS and characterize their neuroanatomical correlates
Pastor et al., 2021 [65]	Neurophysiological characterization of posteromedial hypothalamus in anaesthetized patients	Brain Sciences	Spain	Single-center	To characterize the neurophysiological properties of PMH identified by the best neurophysiological response to electrical stimulation
Torres, et al., 2021 [86]	Deep brain stimulation for aggressiveness: Long-term follow-up and tractography study of the stimulated brain areas	Journal of Neurosurgery	Spain	Single-center	To describe long-term outcomes in patients with PMH DBS and tractography analysis in some of the responders
Escobar Vidarte et al., 2022 [23]	Deep brain stimulation for severe and intractable aggressive behavior	Stereotactic and Functional Neurosurgery	Colombia	Single-center	To report on experience in treating patients with low intelligence quotient and severe and refractory aggressiveness using DBS of PMH
Venetucci Gouveia et al., 2023 [31]	Multi-centre analysis of networks and genes modulated by hypothalamic stimulation in patients with aggressive behaviours	ELife	Colombia, Brazil, Spain	Multi-center	To retrospectively investigate the mechanism of action of PH DBS using a multi-centre dataset imaging analysis

*CH* chronic headache; *CCH* chronic cluster headache;* DBS* deep brain stimulation; *PH* posterior hypothalamus; *PMH* posteromedial hypothalamus

## Results

### An overview of literature search results

**Fig. 1:**
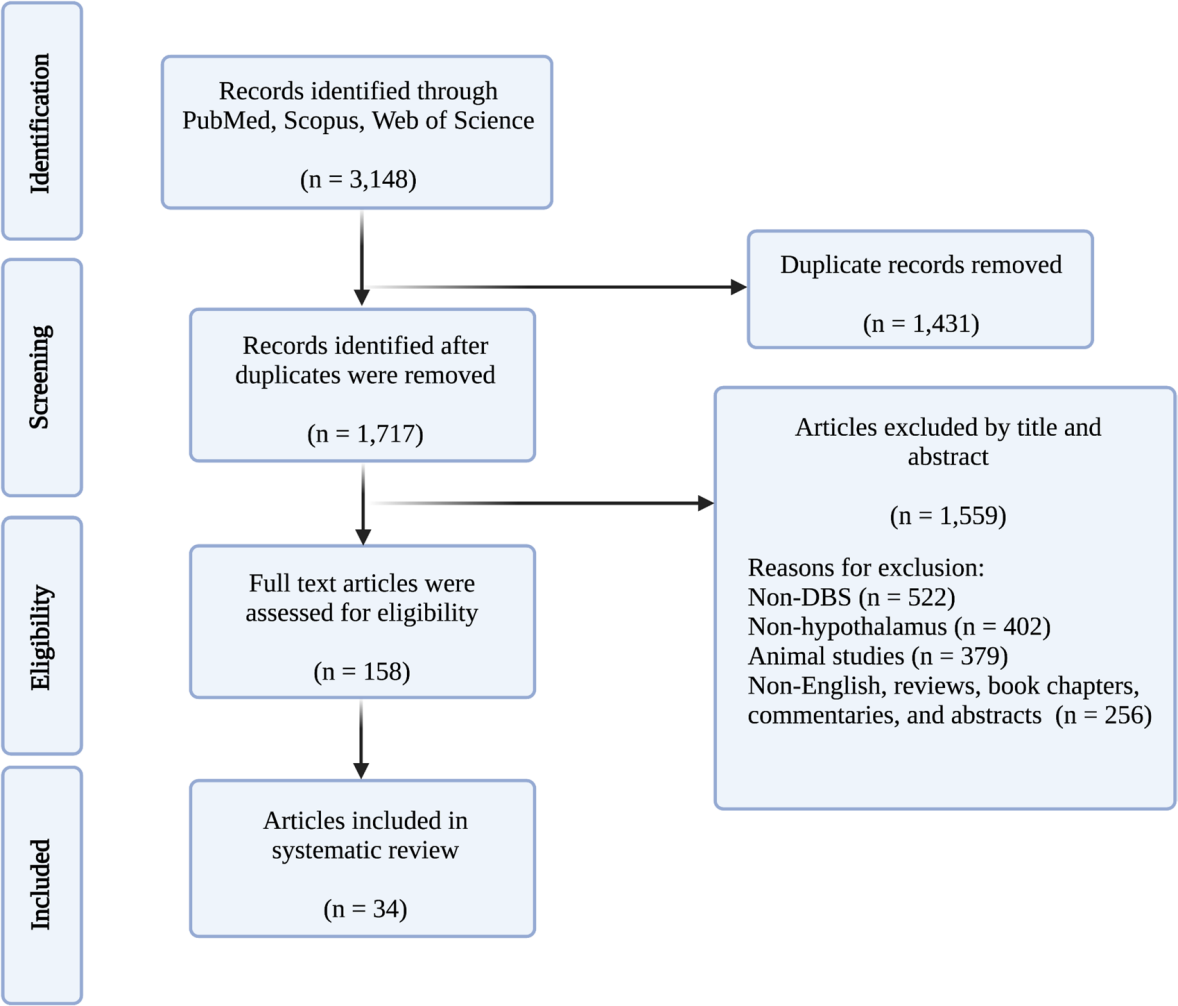
Preferred Reporting Items for Systematic Reviews and Meta-Analyses (PRISMA) flowchart demonstrating search, screen, inclusion, and exclusion process for the current study. DBS, deep brain stimulation

Our search across three databases resulted in 3,148 papers (1,455 from PubMed, 1,042 from Scopus, and 651 from Web of Science). Duplicate removal (*n* = 1,431) yielded 1,717 papers, titles and abstracts which were screened for eligibility. Then, 158 papers were selected for full-text screening, and 34 papers were eligible for the final review (Fig. 1).

An overview of included studies is presented in Table 1 [2, 8–11, 14, 18–20, 23, 25–29, 31, 37, 45–48, 55, 56, 62, 63, 65, 66, 73–75, 77, 82, 86, 87]. Of 34 studies, 24 (70.6%) were from Europe [2, 8, 9, 14, 19, 20, 25, 26, 28, 29, 37, 45–48, 55, 63, 65, 66, 73–75, 86, 87], six (17.6%) were from South America [10, 11, 18, 23, 27, 56], and three (8.8%) were from North America [62, 77, 82]. One (2.9%) multi-center study was conducted in Europe and South America [31].

### Characteristics of patients included in the review

In total, 412 patients were included (Table 2). The largest sample size consisted of 58 (14.1%) patients [62], with the smallest having four (0.97%) patients, each [18, 27, 56, 65, 66, 82]. From 31 (91.2%) studies which specified the gender of participants [2, 8–10, 14, 18–20, 23, 26–29, 37, 45–48, 55, 56, 62, 63, 65, 66, 73–75, 77, 86, 87]. In total, 262 (63.6%) patients were males, and 110 (26.7%) were females. Three (8.8%) studies with 33 (8.0%) patients did not specify the sex of the participants [11, 26, 82]. Thirty-three (8.0%) participants from five (14.7%) studies were pediatrics [10, 23, 31, 56, 86], whereas 303 (73.5%) participants were adults. Three (8.8%) studies did not specify whether participants were adults and/or pediatrics [9, 11, 45]. The highest mean age of participants was 68.5 ± 7.9 years in mild Alzheimer’s disease patients [62], and the lowest one was 15.25 ± 4.6 for chronic refractory aggressiveness [56].

**Table 2:** Characteristics of patients from studies reviewed

Study	Study duration	Total patient number	Sex		Mean age years SD (range)	Adult and / or pediatric		Handedness (L, R) (n, %)
			Male (n, %)	Female (n, %)		Adult (≥ 18)	Pediatric	
Leone et al., 2003 [45]	NS	7	5 (71.4%)	2 (28.6%)	NS (27—63)	7 (100%)	0 (0%)	NS
Franzini et al., 2004 [29]	2000—NS	8	5 (62.5%)	3 (37.5%)	42.2 ± 10.9 (30—63)	8 (100%)	0 (0%)	NS
Schoenen, et al., 2005 [73]	NS	6	5 (83.3%)	1 (16.7%)	46.7 ± 7 (34—53)	6 (100%)	0 (0%)	NS
Leone et al., 2006a [46]	NS	16	14 (87.5%)	2 (12.5%)	43*^1^ (25—70)	16 (100%)	0 (0%)	NS
Leone et al., 2006b [47]	2000—NS	16	14 (87.5%)	2 (12.5%)	43 (NS)	NS	NS	NS
May et al., 2006 [55]	NS	10	9 (90.0%)	1 (10.0%)	43.9 ± 13.2 (25—63)	10 (100%)	0 (0%)	L (0, 0%), R (10, 100%)
Broggi et al., 2007 [14]	7 years (NS)	20	16 (80.0%)	4 (20.0%)	46 ± 12.9 (24—63)	20 (100%)	0 (0%)	NS
Cortelli et al., 2007 [20]	NS	8	7 (87.5%)	1 (12.5%)	34.8 ± 8.8 (24—47)	8 (100%)	0 (0%)	NS
Starr et al., 2007 [82]	NS	4	NS	NS	54.8 ± 9.04 (41—66)	4 (100%)	0 (0%)	NS
Bartsch et al., 2008 [8]	NS	6	4 (66.7%)	2 (33.3%)	40 ± 10.1 (24—55)	6 (100%)	0 (0%)	NS
Cordella et al., 2009 [19]	NS	5	3 (60.0%)	2 (40.0%)	56 ± 5.1 (49—65)	5 (100%)	0 (0%)	NS
Jurgens et al., 2009 [37]	NS	11	10 (90.9%)	1 (9.1%)	46.5 ± 12.4 (26—65)	11 (100%)	0 (0%)	NS
Fontaine et al., 2010a [25]	NS	10	NS	NS	NS (18—65)	10 (100%)	0 (0%)	NS
Fontaine et al., 2010b [26]	May 2005- Jun 2007	11	8 (72.7%)	3 (27.3%)	44.1 ± 5.0 (36—52)	11 (100%)	0 (0%)	NS
Sillay et al., 2010 [77]	NS	5	4 (80.0%)	1 (20.0%)	49 ± 11.0 (38—66)	5 (100%)	0 (0%)	NS
Seijo et al., 2011 [74]	NS	5	4 (80.0%)	1 (20.0%)	47.5 ± 1.95 (45—50)	5 (100%)	0 (0%)	NS
Franzini et al., 2013 [28]	2002—2010	7	6 (85.7%)	1 (14.3%)	35 ± 14.2 (20—64)	7 (100%)	0 (0%)	NS
Leone et al., 2013 [48]	2000—2009	19	15 (78.9%)	4 (21.1%)	42 ± 12.5 (24—70)	19 (100%)	0 (0%)	NS
Torres et al., 2013 [87]	2005—NS	6	4 (66.7%)	2 (33.3%)	28.2 ± 4.8 (17—48)	6 (100%)	0 (0%)	NS
Piacentino et al., 2014 [66]	2004—2006	4	4 (100.0%)	0 (0.0%)	49 ± 12.0 (35—66)	4 (100%)	0 (0%)	NS
Benedetti-Isaac et al., 2015 [10]	2010—2014	5	4 (80.0%)	1 (20.0%)	21.4 ± 6.3 (16—33)	3 (60.0%)	2 (40.0%)	NS
Akram et al., 2017 [2]	NS	7	5 (71.4%)	2 (28.6%)	49.7 ± 6.1 (42—61)	7 (100%)	0 (0%)	NS
Micieli et al., 2017 [56]	NS	4	3 (75.0%)	1 (25.0%)	15.25 ± 4.6 (10—19)	1 (25%)	3 (75%)	NS
Seijo-Fernandez et al., 2018 [75]	NS	15	11 (73.3%)	4 (26.7%)	46.9 ± 7.2 (28—60)	15 (100%)	0 (0%)	NS
Franco et al., 2018 [27]	NS	4	2 (50.0%)	2 (50.0%)	NS (18—28)	4 (100%)	0 (0%)	NS
Nowacki et al., 2019 [63]	11 years (NS)	6	5 (83.3%)	1 (16.7%)	51 ± 7.1 (42—60)	6 (100%)	0 (0%)	NS
Belvis et al., 2020 [9]	Nov 2003—Jun 2018	44	31 (70.5%)	13 (29.5%)	38.3 (NS)	NS	NS	NS
Benedetti-Isaac et al., 2021 [11]	NS	19	NS	NS	18.4 ± 4.0 (NS)	NS	NS	NS
Contreras Lopez et al., 2021 [18]	2017—2019	4	3 (75.0%)	1 (25.0%)	26 ± 2.5 (22—28)	4 (100%)	0 (0%)	NS
Neudorfer et al., 2021 [62]	2007—2019	58	32 (55.2%)	26 (44.8%)	68.5 ± 7.9 (NS)	58 (100%)	0 (0%)	NS
Pastor et al., 2021 [65]	NS	4	2 (50.0%)	2 (50.0%)	32.3 ± 11.0 (22—48)	4 (100%)	0 (0%)	NS
Torres, et al., 2021 [86]	2005—NS	7	5 (71.4%)	2 (28.6%)	31.4 ± 13 (17—51)	6 (85.7%)	1 (14.3%)	NS
Escobar Vidarte et al., 2022 [23]	2009—2019	11	10 (90.9%)	1 (9.1%)	18.9 ± 6.2 (10—31)	5 (45.5%)	6 (54.5%)	NS
Venetucci Gouveia et al., 2023 [31]	NS	33	12 (36.4%)	21 (63.6%)	24.5 ± 10.3 (10—52)	22 (66.7%)	11 (33.3%)*^2^	NS

^*^^1^The reported figure is the median. *^2^The threshold for pediatric patients was specified as17 years old. *NS* not specified; *SD* standard deviation

### Indications for DBS of the hypothalamus

Refractory chronic and chronic cluster headaches were the most common indication for targeting the hypothalamus in DBS reported for 238 (57.8%) patients in 21 (61.8%) studies [2, 8, 9, 14, 20, 25, 26, 29, 37, 45–48, 55, 63, 66, 73–75, 77, 82] (Table 3**)**, followed by aggressive behavior reported in 100 (24.3%) patients in 10 (29.4%) studies [10, 11, 18, 23, 28, 31, 56, 65, 86, 87]. Other indications included mild Alzheimer’s disease (58, 14.1%) [62], trigeminal neuralgia in multiple sclerosis (*n* = 5, 1.2%) [19], Prader-Willi syndrome (4, 0.97%) [27], and atypical facial pain (3, 0.72%) [14].

**Table 3:** Details of hypothalamus deep brain stimulation indications

Study	Diagnosis (n, %)	Disease duration years mean ± SD (range)	Signs and symptoms (n, %)	Medications and other treatments (n, %)
Leone et al., 2003 [45]	Refractory CCH (7, 100%)	NS	NS	Postoperative: methysergide + verapamil (1, 14.3%)
Franzini et al., 2004 [29]	Refractory CCH (8, 100%)	3.9 ± 1.9 (1—7)	Oculofacial autonomic with mild hypersexual and hyperphagia (1, 12.5%)	Corticosteroids, lithium, methysergide, ergotamine, verapamil, beta-blockers, tricyclic antidepressants, valproate, topiramate, gabapentin, melatonin, and NSAIDs (8, 100%). Transnasal endoscopic block of the sphenopalatine ganglion twice (8, 100%). Consecutive radiofrequency trigeminal rhizotomies twice (1, 12.5%)
Schoenen, et al., 2005 [73]	Refractory CCH (6, 100%)	6.7 ± 2.6 (3—10)	NS	Steroids, verapamil, methysergide, lithium and/or ergotamine (6, 100%)
Leone et al., 2006a [46]	Refractory CH (16, 100%)	> 1 year	NS	NS
Leone et al., 2006b [47]	Refractory CCH (16, 100%)	3 (1—10)	NS	NS
May et al., 2006 [55]	Refractory CCH (10, 100%)	NS	NS	Verapamil (4, 40.0%), methysergide (2, 20.0%)
Broggi et al., 2007 [14]	Refractory CCH (16, 80%), SUNCT (1, 5%), atypical facial pain (3, 15%)	3.3 ± 2.3 (1—10)	SUNCT: 2–20 s, severe pain episodes, ipsilateral eyelid edema, eye reddening, nostril obstruction, and large tearing. Atypical facial pain: hypoaesthesia and burning pain (3, 100%)	Preoperative CCH: corticosteroids, lithium, methysergide, calcium channel blockers, antiepileptic drugs, pizotifen, tricyclic, melatonin and NSAIDs (16, 100%). Pain attacks: high dose IV dexamethasone and 2–4 infiltrations of a mixture of local anesthetics and corticosteroids to the ipsilateral sphenopalatine ganglion (16, 100%) Postoperative CCH: verapamil (4, 25.0%), methysergide (2, 12.5%) Preoperative SUNCT: carbamazepine, gabapentin, oral and IV valproate, lamotrigine, topiramate, indomethacin, corticosteroids (methylprednisolone, prednisone), and tramadol Atypical facial pain: radical transmandibular tumor resection (1, 33.3%) and drug therapy with carbamazepine, NSAIDs, tropic anesthetics, and opioids (1, 33.3%), carbamazepine, lamotrigine, and phenytoin (1, 33.3%)
Cortelli et al., 2007 [20]	Refractory CCH (8, 100%)	NS	NS	Preoperative: verapamil (6, 75.0%), valproic acid (2, 25.0%), prednisolone (3, 37.5%), methysergide (1, 12.5%), tramadol (1, 12.5%), lithium (1, 12.5%), ranitidine (2, 25.0%) Postoperative: verapamil (2, 25.0%)
Starr et al., 2007 [82]	Refractory CH (4, 100%)	19.8 ± 10.1 (12—37)	NS	Preoperative: hydrocortisone (1, 25.0%), levetiracetam (1, 25.0%), prednisolone (2, 50.0%), verapamil (1, 25.0%), lithium (1, 25.0%), frovatriptan (1, 25.0%), depakote (1, 25.0%), oxygen (1, 25.0%), sumatriptan (2, 50.0%) Postoperative: hydrocodone (1, 25.0%), levetiracetam (1, 25.0%), verapamil (1, 25.0%), lithium (1, 25.0%), frovatriptan (1, 25.0%), depakote (1, 25.0%), methergine (1, 25.0%), abortive sumatriptan (1, 25.0%)
Bartsch et al., 2008 [8]	Refractory CCH (6, 100%)	7.0 ± 5.2 (2—16)	NS	Preoperative: verapamil (4, 66.7%), rizatriptan (1, 16.7%), oxygen (6, 100%), pregabaline (1, 16.7%), prednisolone (6, 100%), sumatriptan (4, 66.7%), lithium (3, 50.0%), methysergid (2, 33.3%), valporate (2, 33.3%), Ca-antagonist (1, 16.7%), topiramate (3, 50.0%), trimipramine (1, 16.7%), ergotamine (2, 33.3%), triptanes (1, 16.7%), citalopram (1, 16.7%), maprotiline (1, 16.7%), oxycodon (1, 16.7%), lidocaine (1, 16.7%), amitriptyline (1, 16.7%), nortriptyline (1, 16.7%), sertaline (1, 16.7%), NSAIDs (1, 16.7%), indomethacin (1, 16.7%), lamotrigine (1, 16.7%), MST (1, 16.7%), clonazepam (1, 16.7%), oxicodon (1, 16.7%), zopiclon (1, 16.7%) Postoperative: verapamil (2, 33.3%), oxygen (4, 66.7%), sumatriptan (2, 33.3%), opiate (1, 16.7%), lamotrigine (1, 16.7%), clonazepam (1, 16.7%), citalopram (1, 16.7%), topiramate (1, 16.7%)
Cordella et al., 2009 [19]	Refractory and recurrent trigeminal neuralgia in multiple sclerosis (5, 100%)	12 (4—21)	Referred pain in all 3 trigeminal territories (2, 40%), pain in 1st and 2nd divisions (3, 60%)	Analgesic (5, 100%), percutaneous balloon compressions (5, 100%), mitoxantrone, vinorelbine and prednisone (2, 40.0%), termorizothomies (2, 40.0%) and refractory to: carbamazepine, phenytoin, gabapentin, lamotrigine (5, 100%)
Jurgens et al., 2009 [37]	Refractory CCH (11, 100%)	13.7 ± 5.5 (6—25)	NS	Postoperative: verapamil (4, 36.4%), methysergide (1, 9.1%)
Fontaine et al., 2010a [25]	Refractory CCH (10, 100%)	> 3 years	NS	Verapamil and lithium
Fontaine et al., 2010b [26]	Refractory CCH (11, 100%)	12.1 ± 8.7 (3—35)	NS	Preoperative: verapamil (9, 81.8%), lithium (5, 45.5%), prednisolone (1, 9.1%)
Sillay et al., 2010 [77]	CH (5, 100%)	15.0 ± 8.0 (8—30)	NS	Occipital nerve stimulation, verapamil, lithium, divalproex sodium, methysergide, topiramate, gabapentin, NSAIDs including indomethacin, and short-term use of corticosteroids, oxygen, sumatriptan, opiates (5, 100%)
Seijo et al., 2011 [74]	Refractory CCH (5, 100%)	5.5 ± 5.5 (2—15)	NS	Verapamil, topiramate, valproate and lithium (5, 100%). Thermocoagulation or gamma-knife procedures (4, 80.0%), bilateral suboccipital stimulation (1, 20.0%). Oxygen (5, 100%), sumatriptan (4, 80.0%), steroids (5, 100%)
Franzini et al., 2013 [28]	Refractory aggressive behavior and mental retardation (7, 100%)	NS	NS	Chlorpromazine (4, 57.1%), thioridazine (1, 14.3%), clotiapine (2, 28.6%), carbamazepine (1, 14.3%), clonazepam (4, 57.1%), valproate (1, 14.3%), quetiapine (1, 14.3%), bromazepam (1, 14.3%), haloperidol (3, 42.9%), promazine (4, 57.1%), diazepam (1, 14.3%), lorazepam (1, 14.3%)
Leone et al., 2013 [48]	Refractory CCH (19, 100%)	3.3 ± 2.3 (1 −10)	NS	Verapamil, lithium carbonate, methysergide, valproate, topiramate, gabapentin, melatonin, pizotifen, ergotamine, indomethacin, steroids (19, 100%), greater occipital nerve stimulation (5, 26.3%)
Torres et al., 2013 [87]	Refractory aggressive behavior (associated with erethism) (6, 100%)	> 5 years	NS	Lesioning of the bilateral left stria terminalis, anterior cingulum, internal capsule, right PMH, and dorsomedial and intralaminar thalamic nuclei (1, 16.7%). Olanzapine (3, 50.0%), clotiapine (2, 33.3%), clorazepate (3, 50.0%), biperiden (2, 33.3%), zuclopenthixol (1, 16.7%), valporate (2, 33.3%), topiramate (2, 33.3%), risperidone (2, 33.3%), quetiapine (1, 16.7%), propanolol (1, 16.7%), oxcarbazepine (1, 16.7%), lormetazepam (1, 16.7%), lorazepam (1, 16.7%), lithium (1, 16.7%), levopromazine (1, 16.7%), levomepromazine (2, 33.3%), gabapentin (2, 33.3%), flunitrazepam (1, 16.7%), diazepam (1, 16.7%), cyproterone (1, 16.7%), clonazepam (1, 16.7%), citalopram (1, 16.7%), chlorpromazine (1, 16.7%), carbamazepine (1, 16.7%), aripiprazole (1, 16.7%)
Piacentino et al., 2014 [66]	Refractory CH (3, 75%), atypical CH (1, 25%)	9.8 ± 3.6 (8—16)	NS	Verapamil (4, 100%), carbolithium (4, 100%), hydrocortisone (4, 100%)
Benedetti-Isaac et al., 2015 [10]	Aggressive behavior associated with refractory epilepsy (5, 100%)	16.8 ± 3.4 (11—21)	NS	Carbamazepine (5, 100%), valproic acid (3, 60.0%), clobazam (2, 40.0%), clozapine (3, 60.0%), phenobarbital (2, 40.0%), quetiapine (5, 100%), phenytoin (3, 60.0%), clonazepam (4, 80.0%), risperidone (2, 40.0%), divalproate (1, 20.0%), levetiracetam (1, 20.0%), oxcarbazepine (1, 20.0%), lacosamide (1, 20.0%), lamotrigine (1, 20.0%), topiramate (1, 20.0%), levomepromazine (1, 20.0%), lorazepam (1, 20.0%). Behavioral therapy (5, 100%)
Akram et al., 2017 [2]	Refractory CCH (7, 100%)	13.0 ± 8.0 (4—25)	NS	Occipital nerve stimulation (1, 14.3%)
Micieli et al., 2017 [56]	Refractory aggressive behavior (4, 100%)	9.5 ± 3.6 (5—15)	NS	Temporal lobectomy (1, 25.0%)
Seijo-Fernandez et al., 2018 [75]	Refractory CCH (15, 100%)	6.8 ± 4.7 (2—15)	NS	Trigeminal thermocoagulation (6, 40.0%), radiosurgery on trigeminal sensitive root (3, 20.0%), NSAIDs (15, 100%), verapamil (15, 100%), topiramate (14, 93.3%), valproic acid (14, 93.3%), lithium (14, 93.3%), prednisolone (14, 93.3%), oxygen (15, 100%), triptans (15, 100%), thermocoagulation (3, 20.0%), gamma knife (2, 13.3%), occipital nerve stimulation (5, 33.3%) Postoperative: NSAIDs (2, 13.3%), verapamil (5, 33.3%), topiramate (2, 13.3%), oxygen (3, 20.0%), triptans (2, 13.3%), lithium (2, 13.3%)
Franco et al., 2018 [27]	Prader-Willi syndrome (4, 100%)	NS	NS	Sleeve gastrectomy (2, 50.0%), Roux-en-Y gastric bypass (1, 25.0%), growth hormone during childhood (4, 100%), clozapine (1, 25.0%), topiramate (2, 50.0%), periciazine (1, 25.0%), clonazepam (1, 25.0%)
Nowacki et al., 2019 [63]	Refractory CCH (6, 100%)	11.8 ± 7.8 (6—29)	NS	Preoperative: sumatriptan (6, 100%), oxygen (6, 100%), NSAIDs (5, 83.3%), verapamil (5, 83.3%), topiramate (2, 33.3%), carbamazepine (1, 16.7%), prednisolone (4, 66.7%), methysergide (5, 83.3%), lithium (5, 83.3%), pregabalin (3, 50.0%), gabapentin (3, 50.0%)
Belvis et al., 2020 [9]	Refractory CCH (44, 100%)	NS	NS	Radiofrequencies of the sphenopalatine ganglion ipsilateral to pain (9, 100%), bilateral stimulation of the occipital nerves (7, 77.8%). Without Verapamil, corticosteroids, lithium, valproic acid and topiramate (44, 100%), botulinum toxin A (11, 25.0%), ipsilateral occipital nerve blocks with anesthetic and corticosteroids (18, 40.0%)
Benedetti-Isaac et al., 2021 [11]	Refractory aggression behavior with intellectual disability (19, 100%)	NS	NS	NS
Contreras Lopez et al., 2021 [18]	Refractory intermittent explosive disorder (4, 100%)	NS	Self-injurious behavior (4, 100%), hetero-aggressive behavior (3, 75%), hyperphagia (1, 25%), anxiety (1, 25%), hypersexuality (1, 25%), onychophagia (1, 25%)	Valproate sodium (3, 75.0%), olanzapine (1, 25.0%), levomepromazine (2, 50.0%), sertraline (2, 50.0%), escitalopram (1, 25.0%), quetiapine (1, 25.0%), clonazepam (2, 50.0%), carbamazepine (2, 50.0%), clozapine (2, 50.0%), risperidone (1, 25.0%), biperiden (1, 25.0%), behavioral therapy (2, 50.0%)
Neudorfer et al., 2021 [62]	Mild Alzheimer’s disease (58, 100%)	2.2 ± 1.7	NS	NS
Pastor et al., 2021 [65]	Refractory aggressive behavior (4, 100%)	NS	NS	Olanzapine (3, 75.0%), topiramate (2, 50.0%), risperidone (2, 50.0%), levomepromazine (2, 50.0%), gabapentin (2, 50.0%), clorazepate (2, 50.0%), zuclopenthixol (1, 25.0%), valproic acid (1, 25.0%), lithium (1, 25.0%), cyproterone (1, 25.0%), citalopram (1, 25.0%), carbamazepine (1, 25.0%), aripiprazole (1, 25.0%)
Torres, et al., 2021 [86]	Refractory aggressive behavior (7, 100%)	> 5 years	NS	Radiofrequency lesion of anterior cingulum, bilateral left stria terminalis, dorsomedial and intralaminar thalamic nuclei, internal capsule, and right PMH (1, 14.3%), resection of a temporal lobe hematoma and arteriovenous malformation (1, 14.3%), electroconvulsive therapy (1, 14.3%). Olanzapine (4, 57.1%), levomepromazine (3, 42.9%), clorazepate (3, 42.9%), valproate (2, 28.6%), topiramate (2, 28.6%), risperidone (2, 28.6%), gabapentin (2, 28.6%), clotiapine (2, 28.6%), biperiden (2, 28.6%), zuclopentixol (1, 14.3%), valproic (1, 14.3%), simvastatin (1, 14.3%), rivotril (1, 14.3%), quetiapine (1, 14.3%), propranolol (1, 14.3%), propanolol (1, 14.3%), oxcarbazepine (1, 14.3%), mirtazapine (1, 14.3%), lormetazepam (1, 14.3%), lorazepam (1, 14.3%), lithium (1, 14.3%), levopromazine (1, 14.3%), lactulose (1, 14.3%), gabapentine (1, 14.3%), flunitrazepam (1, 14.3%), diazepam (1, 14.3%), cyproterone (1, 14.3%), clonazepam (1, 14.3%), citalopram (1, 14.3%), chlorpromazine (1, 14.3%), carbamazepine (1, 14.3%), aripiprazole (1, 14.3%)
Escobar Vidarte et al., 2022 [23]	Refractory aggressive behavior (11, 100%)	9.2 ± 4.2 (4—18)	NS	Bilateral hypothalamotomy via radiofrequency (1, 9.1%)
Venetucci Gouveia et al., 2023 [31]	Refractory aggressive behavior (33, 100%)	NS	Self-injurious and extreme aggressive behaviors towards others and their surroundings	NS

*CCH* chronic cluster headache; *IV *intravenous; *NS* not specified; *NSAID* non-steroidal anti-inflammatory drug; *PMH* posteromedial hypothalamus; *SD* standard deviation; *SUNCT* short-lasting, unilateral, neuralgiform headache attacks with conjunctival injection and tearing

Twenty studies (58.8%) reported the mean disease duration (Table 3) [2, 8, 10, 14, 19, 23, 26, 29, 37, 47, 48, 56, 62, 63, 66, 73–75, 77, 82]. The longest mean duration of the disease was 19.8 ± 10.1 years for medically refractory CH in four (0.97%) patients [82], whereas the shortest one was 2.2 ± 1.7 years for mild Alzheimer’s disease in 58 (14.1%) patients [62]. The longest disease duration for a single patient was 37 years for medically refractory CH reported by Starr et al. [82], and the shortest one was one year for headache reported in four studies [14, 29, 47, 48]. Ten (29.4%) studies did not report a disease duration [9, 11, 18, 20, 27, 28, 31, 45, 55, 65].

Other patient characteristics, including radiological findings, and medical history, are summarized in Table 4.

**Table 4:** A summary of radiological findings and patient history

Study	Radiological findings (n, %)	Other medical history (n, %)	Other noteworthy patient characteristics (n, %)
Leone et al., 2003 [45]	NS	NS	Mean number of attacks per day: 7
Franzini et al., 2004 [29]	NS	NS	Mean range of attacks per day: 3–7
Schoenen, et al., 2005 [73]	Left intracerebral hemorrhage with ventricular inundation postoperatively (1, 16.7%). Saccular aneurysm in the supracavernous portion of the left carotid artery (1, 16.7%)	None (6, 100%)	Attack frequency per day average was 2–4. 1 (16.7%) patient had 3 suicidal attempts
Leone et al., 2006a [46]	NS	NS	2 (12.5%) patients had chronic CH from the onset, and 14 (87.5%) ones had chronic evolved from episodic
Leone et al., 2006b [47]	NS	NS	Patients had 5–8 attacks per day
May et al., 2006 [55]	NS	NS	Patients had 4–8 attacks per day
Broggi et al., 2007 [14]	None	Steroid-induced leg myopathy (1, 5.0%), myocardial infarction due to triptan overuse (1, 5.0%), right posterior mandibular carcinoma (1, 5.0%), rhinopharynx carcinoma (1, 5.0%)	CCH: 2 (12.5%) chronic from onset and in 14 (87.5%) developed from episodic. 1 (6.3%) attempted suicide twice due to pain severity. None (16, 100%) were able to work, and families were severely impacted. CCH 7 attacks/day on average and a mean duration of 90 min SUNCT: attacks > 100 times/day with a mean of 70 min
Cortelli et al., 2007 [20]	NS	NS	Mean systolic blood pressure: 107 mmHg before and 113 mmHg after DBS. Mean diastolic blood pressure: 64 mmHg before and 67 mmHg after DBS. Mean heart rate: 67 before and 65 after DBS at rest supine
Starr et al., 2007 [82]	NS	NS	Mean numbers of attacks per week: 25.5. Mean duration of attacks: 18.8 min. Mean intensity of attacks: 6.4 (on VAS)
Bartsch et al., 2008 [8]	NS	Arterial hypertension (1, 16.7%), right bundle branch block (1, 16.7%), familial CH (1, 16.7%), migraine without aura (1, 16.7%), depression (2, 33.3%), ventricular ulcer (1, 16.7%), hypothyroid state (1, 16.7%), renal infarction (1, 16.7%), lumbar disc hernia (1, 16.7%), bronchitis (1, 16.7%)	Mean attack frequency per day: 3–5. 1 (16.7%) patient had familial CH. 1 (16.7%) patient was suicidal
Cordella et al., 2009 [19]	Multiple demyelinating lesions in the hemispheric white matter, the internal capsule, the ponto-mesencephalic region, and trigeminal pathways (5, 100%). Linear hyperintensity at the pontine trigeminal root entry zone (5, 100%)	multiple sclerosis (5, 100%)	Patients had multiple sclerosis for an average of 23 years. Preoperative pain was severe and not controlled with medication
Jurgens et al., 2009 [37]	NS	NS	Mean attacks per week: 19.1 ± 13.0 (range: 2—49) Hypothalamus DBS induced cold detection threshold and cold pain threshold in the ophthalmic division of the trigeminal nerve
Fontaine et al., 2010a [25]	None	NS	Patients had at least 3 years of CCH and refractory to various trials of medications to be included in the study
Fontaine et al., 2010b [26]	None	NS	Having daily attacks was part of the inclusion criteria. 5 (45.5%) patients had episodic form, and 6 (54.5%) had chronic form with a mean of 17.8 attacks/week
Sillay et al., 2010 [77]	None	NS	Mean attacks per week: 21.6. Mean intensity: 6.66
Seijo et al., 2011 [74]	None	Hypertension (1, 20.0%), hyperlipidemia (2, 40.0%), type II diabetes (1, 20.0%), osteoporosis (1, 20.0%), glaucoma (1, 20.0%), Cushing’s syndrome (1, 20.0%), ischemic cardiomyopathy (1, 20.0%), vertebral crush (1, 20.0%), bilateral femoral head necrosis (1, 20.0%), adenocarcinoma of mouth (1, 20.0%)	Mean number of attacks/day in the past year: 5
Franzini et al., 2013 [28]	Post-traumatic bilateral damage of the temporomesial structures (1, 14.3%), brain ischemia due to cardiac arrest (1, 14.3%). Bilateral frontal cortical atrophy (1, 14.3%), bilateral temporal porencephaly (1,14.3%)	Refractory generalized multifocal epilepsy (2, 28.6%)	Causes of disruptive behavior: idiopathic (4, 57.1%), perinatal toxoplasmosis (1, 14.3%), post-anoxia (1, 14.3%), post-traumatic (1, 14.3%), congenital (unknown origin) (4, 57.1%). IQ < 20 (4, 57.1%), 30 (2, 28.6%), 40 (1, 14.3%)
Leone et al., 2013 [48]	NS	NS	5—8 attacks daily. 2 (10.5%) patients had CCH from the onset, and 17 (89.5%) had episodic CCH
Torres et al., 2013 [87]	Moderate diffuse cortico-subcortical atrophy + pineal cyst (1, 16.7%), gliotic areas related to previous ops (1, 16.7%), extensive encephalomalacia in the right temporal region with preservation of anterior and posteromedial temporal lobe (1, 16.7%). Bilateral thalamus and left hippocampus hypometabolism (1, 16.7%), hypometabolism of thalamus and hypermetabolism of bilateral frontal cortex and striatum (1, 16.7%), globally reduced brain metabolism + moderate hypometabolism in bilateral parietal cortex with severe hypometabolism in the right amygdala, hippocampus and parahippocampal gyrus (1, 16.7%)	Epilepsy (3, 50.0%), cluster headache (1, 16.7%), perinatal hypoxia (2, 33.3%), obsessive compulsive disorder (1, 16.7%), arteriovenous malformation (1, 16.7%), complex partial seizure (1, 16.7%), developmental delay/infantile autism (5, 83.3%)	Daily bouts of uncontrollable aggressiveness directed against objects, caregivers, and themselves. 4 (66.7%) required restraint measures on a daily basis. 5 (83.3%) had severe sleep disturbances. 2 (33.3%) patients had hyperphagia, and 1 (16.7%) had polydipsia. 4 (66.7%) had severe mental retardation, and 1 (16.7%) had moderate mental retardation
Piacentino et al., 2014 [66]	NS	NS	Average number of attacks per day: 6. Duration of the attacks lasting up to 6 h for atypical CH. All (4, 100%) were unable to have a normal social life and were limited in their working activities
Benedetti-Isaac et al., 2015 [10]	Left Sylvian fissure with increased amplitude and discretely dilated frontal horns ipsilaterally related to atrophy (1, 20.0%), tuberous sclerosis (1, 20.0%), bilateral frontotemporal encephalomalacia mainly on the right side with cerebellar atrophy (1, 20.0%), idiopathic basal ganglia calcifications (1, 20.0%), focal increase of tracer uptake in the left temporal brain lobe (1, 20.0%)	Lennox-Gastaut syndrome (1, 20.0%), congenital strabismus (1, 20.0%)	All (5, 100%) had below-average intelligence: IQ = 50 (3, 60.0%), IQ < 40 (2, 40.0%). Causes of epilepsy: TBI (1, 20.0%), tuberous sclerosis (1, 20.0%), acute meningitis aged 14 (1, 20.0%), symptomatic (2, 40%). Mean age at first seizure: 4.6 years
Akram et al., 2017 [2]	NS	NS	Frequency of attacks: 5 per day. Duration of attacks: 147 min
Micieli et al., 2017 [56]	NS	Sotos syndrome (1, 25.0%), hypothyroidism (1, 25.0%), moderate cognitive compromise (3, 75.0%), epilepsy (3, 75.0%), temporal mesial sclerosis (1, 25.0%), tuberous sclerosis (2, 50.0%), West syndrome (1, 25.0%), severe cognitive compromise (1, 25.0%)	Average months of stimulation: 5.75. Episodes of aggressiveness: 2 (50.0%) patients had innumerable episodes per day before DBS, 1 (25.0%) had 3 episodes and 1 (25.0%) had 10 episodes. Institutionalization: 1 (25.0%) 24 h/day, 1 (25.0%) part-time (1, 25.0%) and 2 (50.0%) not at all
Seijo-Fernandez et al., 2018 [75]	None	Second-branch trigeminal neuralgia (1, 6.7%), mouth adenocarcinoma (1, 6.7%)	Mean attacks/week: 39. Mean pain intensity: 9 out of 10. Mean pain duration: 53 min. Mean time from CCH diagnosis to surgery: 7 years
Franco et al., 2018 [27]	NS	Psychiatric comorbidity: skin picking (1, 25.0%), nail biting (1, 25.0%), aggressive behavior (2, 50.0%), hypersexuality (1, 25.0%), episodes of hypomania (1, 25.0%), psychosis (1, 25.0%), and impulsiveness (1, 25.0%). Hypogonadotropic hypogonadism (4, 100%), hepatic steatosis (1, 25.0%), hyperphagia (2, 50.0%), impulsiveness (1, 25.0%)	Severe obesity (4, 100%). Mean baseline body mass index (BMI): 39.6
Nowacki et al., 2019 [63]	None	NS	Mean attack frequency: 29 attacks per week. Mean attack duration: 43.3 min. Mean attack intensity: 9.7 on VAS
Belvis et al., 2020 [9]	None	NS	4 (44.4%) patients had high-risk suicidal ideation
Benedetti-Isaac et al., 2021 [11]	Cortico-subcortical atrophy (NS)	NS	NS
Contreras Lopez et al., 2021 [18]	NS	Congenital rubella (2, 50.0%), infantile autism (1, 25.0%), drug resistant epilepsy (1, 25.0%), hearing impairment (2, 50.0%), obesity associated with compulsive eating disorder (1, 25.0%), decreased visual acuity (1, 25.0%), congenital intestinal malformation (1, 25.0%)	Severe intellectual disability (4, 100%), impoverished quality of life (4, 100%), high risk for self-injury behavior (4, 100%), institutionalized (2, 50.0%)
Neudorfer et al., 2021 [62]	NS	NS	Patients offered DBS if score 0.5 or 1 on the Clinical Dementia Ratings scale and scores of 12–24 on the Alzheimer’s Disease Assessment Scale Cognitive subscale 11 (ADAS-Cog 11) (58, 100%)
Pastor et al., 2021 [65]	Moderate diffuse cortico-subcortical atrophy (1, 25.0%), pineal cyst (1, 25.0%), normal (2, 50.0%), extensive encephalomalacia in right temporal lobe (1, 25.0%)	Cluster headache (1, 25.0%), epilepsy (2, 50.0%), perinatal hypoxia (1, 25.0%), obsessive compulsive disorder (1, 25.0%), arteriovenous malformation (1, 25.0%), complex partial seizure (1, 25.0%)	Severe mental retardation (2, 50.0%), moderate mental retardation (2, 50.0%)
Torres, et al., 2021 [86]	Gliotic areas from previous surgeries (1, 14.3%), moderate diffuse cortico-subcortical atrophy (1, 14.3%), pineal cyst (1, 14.3%), extensive encephalomalacia in the right temporal region with preservation of some areas of anterior and posteromedial temporal lobe (1, 14.3%), bilateral thalamus and left hippocampus hypometabolism (1, 14.3%), hypometabolism of thalamus and hypermetabolism of bilateral frontal cortex and striatum (1, 14.3%), globally reduced brain metabolism with moderate hypometabolism in the bilateral parietal cortex, severe hypometabolism in the right amygdala, hippocampus and parahippocampal gyrus (1, 14.3%)	Epilepsy (3, 42.9%), infantile autism and developmental delay: hyperkinetic aggressiveness (5, 71.4%), perinatal hypoxia and complicated birth (2, 28.6%), obsessive compulsive disorder (1, 14.3%), bradypsychia (1, 14.3%), organic mental disorder (1, 14.3%), insomnia (1, 14.3%), schizophrenia (1, 14.3%), cluster headache (1, 14.3%), arteriovenous malformation (1, 14.3%), dementia (1, 14.3%)	Their quality of life was severely impaired: DSM-IV Global Assessment of Functioning scale (scores < 21), aggressiveness was very serious according to Maladaptive Behavior Index of the Spanish version of the Inventory for Client and Agency Planning (ICAP) (score < − 40). Institutionalized (1, 14.3%). Severe mental retardation (4, 57.1%), moderate mental retardation (1, 14.3%), binge drinking (1, 14.3%), binge eating (1, 14.3%), hyperphagia (2, 28.6%), severe hetero-aggressiveness (1, 14.3%), disinhibition (2, 28.6%)
Escobar Vidarte et al., 2022 [23]	Atrophy (10, 90.9%), bilateral diffuse cortical dysplasia (1, 9.1%)	Autism (1, 9.1%), epilepsy (3, 27.3%)	Low IQ scores: severe (8, 72.7%) or moderate degree (3, 27.3%) of intellectual disability
Venetucci Gouveia et al., 2023 [31]	Diffusion MRI-based tractography (significant association in the extent of volume of activated tissue connectedness to several areas with clinical benefits, such as areas are related to monoamines production)	Intellectual disability (33, 100%), epilepsy (NS), autism spectrum disorder (NS)	Epilepsy (50.0% pediatric, and 62.0% adult). Autism spectrum disorder (34.0% pediatric, 24.0% adult). Intellectual disability (33, 100%). Intellectual disability was severe in 82.0% of patients (75.0% pediatric, 85.0% adult) and moderate in 18.0% of patients (25.0% pediatric, 15.0% adult)

*CCH* chronic cluster headache; *DBS* deep brain stimulation; *IQ* intelligence quotient; *MRI* magnetic resonance imaging; *NS* not specified; *SUNCT* short-lasting, unilateral, neuralgiform headache attacks with conjunctival injection and tearing; *TBI *traumatic brain injury; *VAS* visual analogue scale

### Hypothalamic targets of DBS and outcomes

**Fig. 2:**
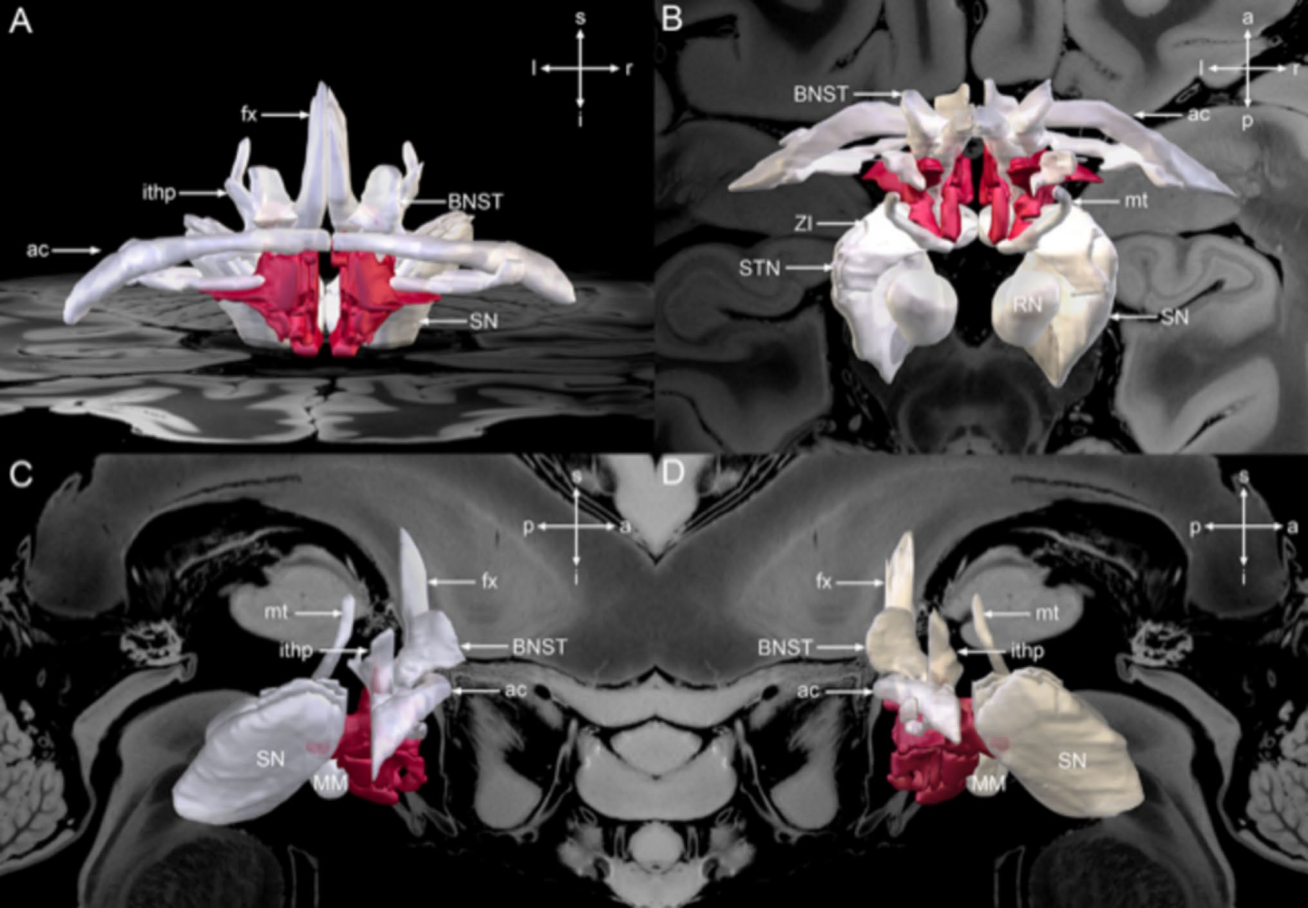
Anatomical relationship between the hypothalamus and surrounding gray and white matter structures. **A** Frontal, **B** superior, **C** right-sided, and **D **left-sided view of the hypothalamus (red) and surrounding structures (gray) overlaid on a 100 μm resolution, 7 T brain scan in Montreal Neurological Institute (MNI) 152 space

Different areas within the hypothalamus were used as target sites for DBS (Table 5). The most common targets were the posterior hypothalamus in 15 (44.1%) studies [8, 9, 14, 19, 20, 26, 28, 29, 31, 37, 46, 56, 63, 66, 82], followed by posteromedial hypothalamus region in seven (20.6%) studies [10, 11, 18, 23, 65, 86, 87], and posteroinferior region in five (14.7%) studies [25, 45, 47, 48, 55] (Figure 2). The posterior hypothalamus was targeted for refractory CH in 133 (32.3%) patients [8, 9, 14, 20, 26, 29, 37, 46, 63, 66, 82], refractory aggressive behavior in 44 (10.7%) patients [28, 56] and refractory trigeminal neuralgia in multiple sclerosis in 5 (1.2%) patients [19]. The posteromedial hypothalamus target was used in 56 (13.6%) patients associated with aggressive behaviors [10, 11, 18, 23, 65, 86, 87]. Sixty-two patients (15.0%) with chronic CH underwent DBS of the posteroinferior hypothalamus [25, 45, 47, 48, 55].

**Table 5:** Patients characteristics of the hypothalamus deep brain stimulation

Study	DBS target (n, %)	Coordinates	Uni (U)/Bilateral (B) (n, %)	Amplitude	Pulse width	Frequency	DBS device
Leone et al., 2003 [45]	Posteroinferior hypothalamus (7, 100%)	NS	U (5, 71.4%), B (2, 28.6%)	NS	NS	NS	NS
Franzini et al., 2004 [29]	PH (8, 100%)	3 mm behind, 5 mm below the MCP, and 2 mm lateral from the midline	U (7, 87.5%), B (1, 12.5%)	0.5—3.8 V	60 μsec	185 Hz	Medtronic 3389
Schoenen, et al., 2005 [73]	Ventroposterior hypothalamus (6, 100%)	2 mm lateral to the midline, 6 mm behind the MCP, and 8 mm below the commissural plane	U (6, 100%)	3.28 V	60 μsec	185 Hz	Medtronic 3389–40
Leone et al., 2006a [46]	PH (16, 100%)	NS	U (14, 87.5%), B (2, 12.5%)	4–5 V	60 μsec	180 Hz	NS
Leone et al., 2006b [47]	Posteroinferior hypothalamus (16, 100%)	5 patients: 6 mm behind the MCP, 8 mm below the mid commissural plane, and 2 mm lateral to the midline 11 patients: 3 mm behind, 5 mm below the MCP, and 2 mm lateral to the midline	U (14, 87.5%) B (2, 12.5%)	2.4 V	60 μsec	180 Hz	NS
May et al., 2006 [55]	Posteroinferior hypothalamic gray cortex (10, 100%)	NS	NS	2.4 V	60 μsec	180 Hz	Medtronic
Broggi et al., 2007 [14]	PH (20, 100%)	3 mm behind, 5 mm below the MCP, and 2 mm lateral from the midline	U (18, 90.0%) B (2, 10.0%)	2.6 V (CH: 2.4 V, AFP: 1.3 V, SUNCT: 1.8 V)	60 μsec	180 Hz	Medtronic 3389
Cortelli et al., 2007 [20]	PH (8, 100%)	3 mm behind, 5 mm below the MCP, and 2 mm lateral to the midline	U (8, 100%)	2.1 V	67.5 μsec	176.3 Hz	Soletra 7426 (3, 37.5%), Soletra NFW625261 (1, 12.5%), Soletra NFW625247 (1, 12.5%), Soletra NFW625255 (1, 12.5%), Kinetra NFD62451 (1, 12.5%), Kinetra NFD624570 (1, 12.5%)
Starr et al., 2007 [82]	PH (4, 100%)	2 mm lateral, 3 mm posterior, and 5 mm inferior to the MCP	U (4, 100%)	1—3 V	60 μsec	185 Hz	Medtronic 3387
Bartsch et al., 2008 [8]	PH (6, 100%)	2 mm lateral, 3 mm posterior and 5 mm inferior to the mid anterior commissure—posterior commissure line	NS	3.7 V	60 μsec	156 Hz	Medtronic 3387 (5, 83.3%), Medtronic 3389 (1, 16.7%)
Cordella et al., 2009 [19]	PH (5, 100%)	3 mm behind, 5 mm below the MCP, and 2 mm lateral to the midline	NS	1—2 V	60 μsec	180 Hz	Medtronic 3389
Jurgens et al., 2009 [37]	PH (11, 100%)	NS	U (11, 100%)	2.3 V	NS	NS	NS
Fontaine et al., 2010a [25]	Posteroinferior hypothalamus (10, 100%)	2 mm lateral, 3 mm posterior and 5 mm below the MCP	U (10, 100%)	2.26 V	60 μsec	185 Hz	Medtronic 3389
Fontaine et al., 2010b [26]	PH (11, 100%)	3 mm posterior, 5 mm below the MCP, and 2 mm lateral to the midline	U (11, 100%)	1—2.8 V	60 μsec	185 Hz	Medtronic 3389
Sillay et al., 2010 [77]	Border zone between PH, anterior periventricular gray matter, and subparafacicular nucleus within the inferomedial thalamus (5, 100%)	2 mm lateral to the midline, 5 mm inferior to the axial plane containing anterior and posterior commissures, and 3 mm posterior to the MCP	NS	1—3 V	60 μsec	185 Hz	Medtronic 3387
Seijo et al., 2011 [74]	Posterolateral hypothalamus (5, 100%)	4 mm from the third ventricle wall, 2 mm from behind the MCP and 5 mm from under the intercommissural line 4.83 mm lateral to the third ventricle wall, 1.40 mm behind the MCP and 4.16 mm below the intercommissural line	U (5, 100%)	2.87 V	75 μsec	130 Hz	Medtronic 3389
Franzini et al., 2013 [28]	PH (7, 100%)	3 mm behind, 5 mm below the MCP, and 2 mm lateral from the midline. 2 mm posterior to the interpeduncular point instead of 3 mm posterior to the MCP	B (7, 100%)	1—3 V	60—90 μsec	185 Hz	Medtronic 3389
Leone et al., 2013 [48]	Posteroinferior hypothalamus (19, 100%)	6 mm behind the MCP, 8 mm below the midcommissural plane, and 2 mm lateral to the midline. For patients 6 onwards: 3 mm behind, 5 mm below the MCP, and 2 mm lateral to the midline	U (15, 79.0%), B (4, 21.0%)	2.8 V	60 μsec	180 Hz	Medtronic 3389
Torres et al., 2013 [87]	PMH (6, 100%)	2 mm lateral to the wall of the third ventricle, at the MCP, and 2 mm inferior to the anterior commissure—posterior commissure line	U (1, 20.0%), B (4, 80.0%)*	2 V	143 μsec	153 Hz	Medtronic 3389
Piacentino et al., 2014 [66]	PH (4, 100%)	3 mm posterior to the midpoint of the anterior commissure—posterior commissure line, 2 mm lateral to the wall of the third ventricle, and 5 mm inferior to the midcommissural line	U (4, 100%)	2.3 V	60 μsec	171 Hz	NS
Benedetti-Isaac et al., 2015 [10]	PMH (5, 100%)	2 mm lateral, 3 mm posterior, and 5 mm inferior to the MCP	B (5, 100%)	2.7 V	90 μsec	185 Hz	Medtronic 3389
Akram et al., 2017 [2]	Trigemino-hypothalamic tract	Original targets were 2 mm lateral to the midline, 6 mm behind the MCP, and 8 mm below the anterior commissural—posterior commissural line. The target was later modified to 2 mm lateral to the midline, 3 mm posterior, and 5 mm below the MCP	U (5, 71.4%), B (2, 28.6%)	3.3 V	NS	NS	NS
Micieli et al., 2017 [56]	PH (4, 100%)	1 mm behind the MCP and 5 mm below the anterior commissural-posterior commissural line and 2 mm lateral to the lateral wall of the third ventricle	B (4, 100%)	NS	NS	NS	NS
Seijo-Fernandez et al., 2018 [75]	Mamillotegmental fasciculus (15, 100%)	4 mm lateral to the third ventricular wall and 2 mm behind and 5 mm below the intercommissural point	U (15, 100%)	2.2 mA	107.7 μsec	115.3 Hz	Medtronic 3389 (10, 66.7%), St Jude 6149 (1, 6.7%), Boston Scientific octopolar electrode (4, 26.7%)
Franco et al., 2018 [27]	Lateral hypothalamic area (4, 100%)	8.3 mm lateral, 5.8 mm anterior, and 7.3 mm inferior to the MCP	B (4, 100%)	2.75 mA	180.3 μsec	62.5 Hz	St Jude Medical 6149
Nowacki et al., 2019 [63]	PH (6, 100%)	Lateral 3.0 mm (range, 1 mm—7.4 mm), anteroposterior 7.8 mm (range, 3.9 mm—10.7 mm), and vertical 1.9 mm (range, 4.7 mm—0 mm)	U (4, 66.7%), B (2, 33.3%)	2 V	90 ms	180 Hz	Medtronic 3389
Belvis et al., 2020 [9]	PH (9, 100%)	NS	U (9, 100%)	NS	NS	NS	NS
Benedetti-Isaac et al., 2021 [11]	PMH (19, 100%)	NS	NS	NS	NS	NS	NS
Contreras Lopez et al., 2021 [18]	PMH (4, 100%)	2 mm lateral to the lateral wall of the third ventricle, 3 mm posterior and 5 mm inferior to the mid-commissural anterior commissure-posterior commissure point	B (4, 100%)	1.55 mA	85 μsec	163 Hz	Boston Scientific
Neudorfer et al., 2021 [62]	Different regions of the hypothalamus (58, 100%)	2 mm anterior to the post-commissural fornix, 5 mm from the midline and above the dorsal surface of the optic tract	B (58, 100%)	5.2 V	90 μsec	130 Hz	Medtronic 3387
Pastor et al., 2021 [65]	PMH (4, 100%)	Sano’s triangle (X = 2 mm, Y = 0 mm, Z = − 2 mm)	U (1, 25.0%), B (3, 75.0%)	NS	NS	NS	Quadripolar DBS electrode
Torres, et al., 2021 [86]	PMH (7, 100%)	X = 2 mm lateral to the wall of the third ventricle, and Y = 0 mm and Z = − 2 mm with respect to the MCP	U (3, 42.9%), B (4, 57.1%)	NS	450 μsec	15–185 Hz	Medtronic 3389, St. Jude Medical, Libra
Escobar Vidarte et al., 2022 [23]	PMH (11, 100%)	2 mm lateral to the midline, 3 mm behind the MCP, and 5 mm below it	B (11, 100%)	1 V	60 μsec	180 Hz	Medtronic 3389
Venetucci Gouveia et al., 2023 [23]	PH (33, 100%)	MNI152 space at the coordinates X = 7.5, Y = −15, Z = −6.5, and Talairach-Tournoux space at coordinates X = 6.5, Y = −16, Z = −1.5	U (3, 9.1%), B (30, 90.9%)	Right hemisphere: 2.45 ± 1.76 V. Left hemisphere: 3.17 ± 1.26 V	Right hemisphere: 219.25 ± 172.46 µsec. Left hemisphere: 142.33 ± 95.48 µsec	Right hemisphere: 166.25 ± 18.87 Hz. Left hemisphere:171.67 ± 18.93 Hz	Medtronic 3387, Medtronic 3389, Boston Vercise

*AFP* atypical facial pain; *B* bilateral; *CH* chronic headache; *MCP* mid-commissural point; *NS* not specified; *PH* posterior hypothalamus; *PMH* posteromedial hypothalamus; *SUNCT* short-lasting, unilateral, neuralgiform headache attacks with conjunctival injection and tearing; *U* unilateral

^*^No data on the patient who died from stroke

Eleven (32.3%) studies with 77 (18.7%) patients had unilateral-only stimulation [9, 20, 25, 26, 37, 66, 74, 75, 82], whereas seven (20.6%) studies containing 93 (22.6%) patients had bilateral-only DBS [10, 18, 23, 27, 28, 56, 62]. In eleven (32.4%) studies which had a mixture of stimulation lateralities, 75 (23.9%) patients received unilateral, and 53 (12.9%) patients received bilateral DBS [2, 14, 23, 29, 45–48, 63, 86, 87]. In total, 152 (36.9%) patients underwent unilateral DBS, whereas 146 (35.4%) patients had bilateral DBS. Five (14.7%) studies with 45 (10.9%) patients did not specify the laterality of the stimulation [8, 11, 19, 55, 77] (Table 5).

An overview of patients’ outcomes is shown inTable 6. Overall, 213 (51.7%) patients across 29 (85.3%) studies reported outcome improvements [2, 8–11, 18, 20, 23, 25, 26, 28, 29, 31, 37, 45–48, 55, 56, 63, 66, 73–75, 77, 82, 86, 87], of whom 126 patients (30.6%) were treated for headache and 68 patients (16.5%) were treated for aggressive behavior.

**Table 6:** A summary of efficacy and main outcomes reported

Study	Patients with positive outcome after DBS (n, %)	Improvement reported on scale	Efficacy assessment	Efficacy assessment scale	Main outcome reported
Leone et al., 2003 [45]	6 (85.7%)	NS	Pain relief	NS	6 (85.7%) patients were pain-free without a need for pharmacological therapy. 1 (14.3%) patient experienced attacks again after an 18-month pain-free period. 4 (57.2%) patients experienced the reappearance and disappearance of pain attacks by switching off and turning on the stimulator, respectively. There was no change in heart rate, blood pressure, electrolyte balance, hormone levels, and behavior in all patients
Franzini et al., 2004 [29]	8 (100%)	> 20% (at least)	Pain relief	NS	All patients had complete pain relief that continued until their most recent follow-up. 3 (37.5%) patients were pain-free without medication. 5 (62.5%) patients had episodic attacks (less than 5/month) with low doses of methysergide or verapamil which were ineffective preoperatively. Overall, there was a progressive drop in the number of daily attacks until disappearance over an average period of 4.4 weeks
Schoenen, et al., 2005 [73]	3 (50.0%)	NS	Reduction in pain attack frequency and intensity	Self-report	2 (33.3%) patients were pain-free, 1 (16.7%) had fewer than 3 attacks per month, and 1 (16.7%) had transient remissions. None were taking prophylactic medications. 4 (66.7%) patients showed improvements. The mechanism of pain relief is not due to a hormonal or analgesic effect. 1 (16.7%) patient died to due implant-induced cerebral hemorrhage
Leone et al., 2006a [46]	25 out of 108 attacks (23.1%)	> 50%	Reduction in pain intensity	VAS	20 min of stimulation (54 monopolar and 54 bipolar stimulation) resulted in pain resolution in 108/136 (79.4%) attacks. In 17/108 (16.0%) treated attacks, the improvement was total pain resolution. Efficacy did not differ between monopolar and bipolar stimulation
Leone et al., 2006b [47]	13 (81.3%)	NS	Pain-free or major reduction in pain	NS	10 (62.5%) patients were pain-free, 3 (18.7%) patients were almost pain-free, and the other 3 (18.7%) had improvements with persistent attacks but reduced frequency, intensity, and duration. 4 (25.0%) patients used prophylaxis to control attacks. The average time to observe benefits was 42 days
May et al., 2006 [55]	10 (100.0%)	NS	Reduction in headache frequency	NS	All (10, 100%) patients improved in their headache frequency. None of the patients experienced any adverse side effects
Broggi et al., 2007 [14]	14 (70.0%)	NS	Percentage of total days free of pain	NS	After 5 years, the number of pain-free days improved from 1%–2% to 71%. The patient with SUNCT has complete pain relief. DBS was not effective in 3 (15.0%) patients with atypical facial pain
Cortelli et al., 2007 [20]	6 (75.0%)	NS	Pain-free	NS	DBS did not result in changes in systolic and diastolic blood pressure, heart rate, and cardiovascular reflexes. DBS of PH resulted in increased sympathoexcitatory drive on the cardiovascular system during the head-up tilt test. 6 (75.0%) patients were completely pain-free, but 2 (25.0%) required prophylactic low doses of verapamil
Starr et al., 2007 [82]	2 (50.0%)	> 50%	Reduction in headache frequency or intensity or both	VAS	DBS resulted in > 50% reduction in headache intensity or frequency in 2 (50.0%) patients at 1-year
Bartsch et al., 2008 [8]	3 (50.0%)	30–90%	Reduction in headache attack frequency	VAS, HIT, HDI, BDI, SF-36, HDS	DBS of hypothalamus resulted in 50.0% success after a mean follow-up of 17 months
Cordella et al., 2009 [19]	5 (100%)	NS	Reduction in the BNI scale	Pain BNI scale	V1 pain was controlled after 1 to 4 years follow-up, whereas the recurrence of pain in V2 and V3 trigeminal branches needed repeated thermorhizotomies to control pain in 2 (40.0%) patients after 2 years of follow-up
Jurgens et al., 2009 [37]	5 (45.5%)	NS	Pain free	NS	DBS of the PH is specific for cluster headache and affects certain aspects of pain perception. Compared to healthy controls, the DBS group had increased pain thresholds at the first trigeminal branch on the stimulated side (*p* = *0.015*) as well as cold detection thresholds compared to non-implanted cluster headache patients (*p* < *0.05*)
Fontaine et al., 2010a [25]	5 (50.0%)	> 50%	Reduction in the weekly frequency of attacks	NS	Failure of DBS in CH can be due to factors unrelated to electrode misplacement. DBS may modulate either a local cluster generator or have a non-specific effect on anti-nociceptive systems. Functional and anatomical differences between patients can explain heterogeneous results
Fontaine et al., 2010b [26]	6 (54.5%)	> 50%	Reduction in the weekly frequency of attacks	SF-12, Likert scale for intensity, HAD	Randomized phase findings do not support the efficacy of DBS in refractory CCH, but open phase backs up long-term efficacy in > 50% patients, without high morbidity. Discrepancy justifies additional controlled studies
Sillay et al., 2010 [77]	3 (60.0%)	> 50%	Reduction in headache frequency, intensity, or both	VAS	DBS resulted in 17–61% improvement in headache attacks on 6–12 months follow-up
Seijo et al., 2011 [74]	5 (100%)	52–100%	Reduction in headache attack frequency	NS	All (5, 100%) patients were pain-free 1–2 weeks postoperatively but needed 54 days on average for parameter optimizations. After 33 months of follow-up, 2 (40.0%) patients were 100% pain-free, 2 (40.0%) had > 90% reduction in attack frequency and 1 (20.0%) had 52% reduction. DBS is efficacious in refractory CCH with a slightly modified hypothalamic target to avoid the lateral ventricle wall and extend the stimulated brain area and decrease the likelihood of hemorrhagic complications
Franzini et al., 2013 [28]	6 (85.7%)	37.5–100%	Reduction in aggression and disruptive bouts with simplification of management	OAS	DBS of the PH is an effective treatment for patients affected by mental retardation with drug-refractory aggressive behavior
Leone et al., 2013 [48]	12 (70.6%)	NS	Pain-free	NS	Long follow-up demonstrates that hypothalamic stimulation for refractory CH produces lasting improvement in many patients. Stimulation is well tolerated for many years. After 3–4 years, a persistent almost pain-free condition can be maintained when stimulation is off, and tolerance can occur after improvement. 5 patients (26.3%) did not improve, 4 (21.1%) of whom had bilateral CH, and 3 (15.8%) developed tolerance after experiencing relief for 1–2 years. Bilateral CH is a predictor of poor DBS outcome
Torres et al., 2013 [87]	5 (83.3%)	46.8%	Reduction in aggressiveness score	ICAP	5 (83.3%) patients with pathological aggressiveness had a reduction of their violence after PMH DBS, without significant adverse effects. Sleep patterns became more regular in 4 (66.7%) patients, and binge eating and polydipsia ceased in 1 (16.6%) patient. A 30% reduction in seizure frequency was reported in 1 of the 3 patients who had epilepsy. 1 (16.7%) patient had a worsening of a previous headache
Piacentino et al., 2014 [66]	4 (100%)	44.4%	Reduction in pain and the number of attacks	VAS	There is long-lasting pain reduction and frequency improvement in the patients’ symptoms. DBS can restore important aspects of the normal daily life of CH patients
Benedetti-Isaac et al., 2015 [10]	5 (100%)	33–100% aggression reduction. 89.6% reduction in seizure	Reduction in seizure frequency and aggression	OAS	DBS of the PH is a safe and effective procedure for the treatment of refractory epilepsy associated with refractory aggressive behavior
Akram et al., 2017 [2]	6 (85.7%)	76 ± 33% headache load, 46 ± 41% attack severity, 58 ± 41% headache frequency, and 51 ± 46% in attack duration	> 30% reduction in headache attacks	Verbal rating scale for headache	Greatest reduction in headache attacks achieved by DBS of an area centered on 6 mm lateral, 2 mm posterior, and 1 mm inferior to the midcommissural point of the third ventricle
Micieli et al., 2017 [56]	4 (100%)	78–97% on MOAS	Reduction in aggressive behavior	MOAS, QOLS	Microelectrode recordings during PH DBS can provide mechanisms to identify the PH target and surrounding structures to be avoided (e.g., the red nucleus). The effect of the anesthetic administered should be considered as well. PH DBS may be an effective treatment for aggression
Seijo-Fernandez et al., 2018 [75]	15 (100%)	60%	Reduction in the number of weekly headache attacks, pain intensity, and duration	NS	DBS is a safe and useful procedure for the treatment of refractory CCH; the improvement was higher than those found in other series. Larger series targeting those fasciculi with a longer follow-up are needed
Franco et al., 2018 [27]	0 (0.0%)	9.6% increase in weight, 5.8% increase in body mass index, 8.4% increase in abdominal circumference, 4.2% increase in neck circumference, 5.3% increase in body fat percentage, and 0.0% change in calorimetry	Reduction in anthropometric measures (weight, body mass index and abdominal and neck circumference), bioimpedanciometry, and calorimetry)	NS	DBS of the lateral hypothalamus is largely ineffective for the treatment of obesity. Two (50.0%) patients developed stimulation-induced manic symptoms, and DBS discontinuation controlled this symptom in 1 (25.0%) patient
Nowacki et al., 2019 [63]	6 (100%)	93%	Reduction in headache attack frequency, duration and intensity	NPS, QOLS, SF-36, VAS	There is long-term effectiveness of DBS for CCH which suggests that the neuroanatomical substrate of DBS-induced headache relief is probably not restricted to the PH but involves a wider area
Belvis et al., 2020 [9]	8 (88.9%)	70.7%	50% reduction in the number of daily attacks after 3 months	NPS	The sequential application of the 3 procedures worked in reversing refractory CCH in the majority of patients with acceptable surgical complications
Benedetti-Isaac et al., 2021 [11]	NS	NS	Reduction in OAS scores	OAS	A clear reduction in aggressive behaviors occurred and remained constant up to 18 months of follow-up. DBS is a successful treatment option for patients with refractory aggression, proving to be effective and safe. Longer follow-up studies are required to confirm the time in which aggressive behavior stabilizes and flattens
Contreras Lopez et al., 2021 [18]	4 (100%)	36.7—58.4%	Reduction in OAS scores, SF-36 changes, changes in neurotransmitter concentration in the PH, measured by intraoperative microdialysis under high-frequency stimulation	OAS, SF-36	A marked increase in the level of gamma-aminobutyric acid and glycine was observed during intraoperative stimulation. An average of 50.0% improvement in aggression was observed
Neudorfer et al., 2021 [62]	NS	NS	NS	NS	Acute effects of hypothalamic region stimulation to distinct tracts and nuclei within the hypothalamus were localized and the wider diencephalon providing insights that may guide future neuromodulation studies
Pastor et al., 2021 [65]	NS	NS	NS	NS	Neurophysiological properties of the hypothalamus are specific, and PMH can be identified during microelectrode recording by the type of cells, presence of atypical extracellular action potentials and the pattern of discharge
Torres, et al., 2021 [86]	5 (71.4%)	38.3%	Reduction in ICAP aggression score	ICAP	5 (71.4%) patients with refractory severe aggressiveness treated with bilateral PMH DBS showed a significant long-lasting improvement. The areas responsible for the effect may be PMH, ventral tegmental area, dorsal longitudinal fasciculus, and medial forebrain bundle
Escobar Vidarte et al., 2022 [23]	9 (81.8%)	63–69%	Reduction in MOAS	MOAS	9 (81.8%) patients had a significant decrease in aggressive behavior intensity from MOAS average value of 50.5 preoperatively to 18.7 postoperatively. 4 years of follow-up showed a 63% average improvement. Deterioration of symptoms following depletion of the pulse generator was recovered after battery replacement. DBS of the PMH may be a safe and effective means to improve severe and refractory aggressive behavior in patients with long-term intellectual disabilities
Venetucci Gouveia et al., 2023 [31]	30 (90.9%)	> 30%	> 30% reduction in MOAS, OAS, ICAP	MOAS, OAS, ICAP	Pediatric patients (≤ 17 years of age) demonstrated greater symptom improvement compared to the adult group (93% vs 66%). However, there were no differences between males and females (75.57% ± 31.13%)

*BDI* Beck depression inventory; *BNI* Barrow Neurological Institute; *CCH* chronic cluster headache; *CH* chronic headache; *DBS* deep brain stimulation; *HDI* headache disability inventory; *HDS* Hamilton depression score; *HIT* headache impact test; *ICAP* inventory for client and agency planning; *MOAS* modified overt aggression scale; *NPS* numeric pain scale; *OAS* overt aggressive scale; *QOLS* quality of life scale; *SF-36*, short form; *SUNCT* short-lasting unilateral neuralgiform headache attacks with conjunctival injection and tearing; *VAS* visual analogue scale

Surgical details of DBS are shown in Table 7. Overall, 25 (73.5%) studies reported complications [8, 10, 11, 14, 18, 19, 23, 25–29, 56, 62, 63, 65, 66, 74, 77, 82, 86, 87], most of which were associated with higher voltage stimulations, for example, conjugated ocular deviation with extreme verbal responses above 4 V stimulation in eight patients, [29], diplopia and dizziness above 1.5 V stimulation [47, 73], and 2 V stimulation44 in most patients and vertigo at 4 V stimulation [66]. The follow-up length ranged from 3 months [45] to 144 months [86], in 29 (85.3%) studies which specified the follow-up length. In one study [73], one patient (0.24%) died soon after the operation due to intracerebral hemorrhage induced by the implant, indicating the risk of hemorrhage.

**Table 7:** Surgical details and complications of DBS

Study	Complications and side effects (n, %)	Follow-up duration
Leone et al., 2003 [45]	None	3—33 months
Franzini et al., 2004 [29]	Higher than 4 V stimulation resulted in conjugated ocular deviation with extreme verbal responses (8, 100%), and decreased food intake (1, 12.5%)	2—26 months
Schoenen, et al., 2005 [73]	Diplopia and dizziness above 1.5 V stimulation (6, 100%), intraoperative panic sensation with polypnoea, tachycardia, and moderate hypertension (1, 16.7%), moderate hypertension (160/120 mmHg) and attack that needed 1 mg IV dihydroergotamine. Comatose after 5 h with bilateral mydriasis. Death 3 days later due to implant-induced (1, 16.7%)	14.5 months
Leone et al., 2006a [46]	General malaise, sweating, dizziness, diplopia, other visual disturbances (NS)	NS
Leone et al., 2006b [47]	Small, asymptomatic hemorrhage into the third ventricle, but disappeared 1 day later (1, 6.3%). Transient voltage-dependent (> 4 V) diplopia (required a few days for complete resolution) in most patients. 15 (93.8%) patients with long follow-up lost weight with 3 months postoperative weight loss of 3 kg on average due to steroid withdrawal	23 months (mean)
May et al., 2006 [55]	None	1 year (mean)
Broggi et al., 2007 [14]	Stimulation beyond 4 V resulted in conjugated ocular deviation with verbal reports (20, 100%). Postoperative CT: mild, not symptomatic, hemorrhage of the posterior wall of the third ventricle (1 CCH patient, 6.3%). CCH: asymptomatic orthostatic hypotension triggered by stimulation (4, 100%), deep infection with subsequent electrode removal (excluded). Need of electrode replacement (1, 6.3%), electrode replacement due to cranial migration (1, 6.3%). SUNCT: stimulation beyond 1.4 V resulted in difficulties in conjugated eye movements. Atypical facial pain: stimulation beyond 3 V resulted in dizziness and oculomotor symptoms (3, 100%). Electrode misplacement (1, 5.0%)	5 years
Cortelli et al., 2007 [20]	4—5 V stimulation resulted in general malaise, generalized sweating, dizziness and diplopia but did not change blood pressure or heart rate (8, 100%)	106.4 days (mean)
Starr et al., 2007 [82]	5 min intraoperative transient ischemic attack (1, 25.0%), ophthalmoplegia at 10 V and 4.5 V (2, 50.0%), double vision at 5 V (1, 25.0%), skew deviation at 1.5 V (1, 25.0%), sense of impending doom/feeling drugged at 5 V and 3 V respectively (2, 50.0%), reversible dysphoria (2, 50.0%), sense of dizziness or warmth at > 4 V (4, 100%)	1 year
Bartsch et al., 2008 [8]	Technical side effects: tense feeling at connection cable (1, 16.7%), voltage-dependent side effects: double vision at 1—4 V (5, 83.3%), arterial hypertension at 3 V (1, 16.7%), vertigo at 1—4 V (4, 66.7%), panic at 4 V (1, 16.7%), intraoperative cluster attack in test stimulation at 1 V, 130 Hz, aborted by oxygen (1, 16.7%)	17 months (mean)
Cordella et al., 2009 [19]	None	41 months (mean)
Jurgens et al., 2009 [37]	NS	NS
Fontaine et al., 2010a [25]	NS	1 year
Fontaine et al., 2010b [26]	Adverse event related to surgery: superficial infection 1 (9.1%), neck pain along lead 1 (9.1%). Adverse effects related to test stimulation: oculomotor changes 4 (36.4%), loss of consciousness with hemiparesia, 1 (9.1%). Adverse effects in ON period: mild hunger increase 3 (27.3%), mild hunger decrease 1 (9.1%), mild libido decrease 2 (18.2%). Adverse effects in OFF period: mild hunger increase 2 (18.2%), mild hunger decrease 1 (9.1%), mild thirst increase 1 (9.1%), mild thirst decrease 1 (9.1%), mild libido decrease 1 (9.1%), increased testosterone 1 (9.1%), shorter menstrual cycle 1 (9.1%). Adverse effects in chronic phase: severe micturition syncope 1 (9.1%), change in postural blood pressure 1 (9.1%), moderate weight increase 1 (9.1%), mild hunger increase 1 (9.1%), mild hunger decrease 1 (9.1%), mild libido decrease 1 (9.1%), increased testosterone level 1 (9.1%)	1 year
Sillay et al., 2010 [77]	Intraoperative transient ischemic attack resolved in 5 min (1, 20.0%)	10.8 months (mean)
Seijo et al., 2011 [74]	Permanent miosis and euphoria (3, 60.0%). Occasional dizziness (3, 60.0%), blurring vision/diplopia (2, 40.0%), concentration difficulties (1, 20.0%), cervical dystonia (1, 20.0%), generalized headache (1, 20.0%) and increase in appetite (1, 20.0%) with stimulation threshold increases. Attacks reappeared in 2 (2, 40.0%) patients due to cable rupture. After 18 months, 1 patient (20.0%) had reappearance of daily cluster headache attacks due to a rupture of intracerebral electrode and resolved by a new electrode implantation	33 months (mean)
Franzini et al., 2013 [28]	Voltage-dependent internal gaze deviation (7, 100%). Electrode removal due to skin erosions but reimplanted (1, 14.3%)	4.9 years (mean)
Leone et al., 2013 [48]	Electrode displacement (2, 10.5%), infection of electrode (3, 15.8%), infection of the generator (1, 5.3%), electrode malpositioning (1, 5.3%), transient asymptomatic third ventricle haemorrhage (1, 5.3%), slight muscle weakness on 1 side (1, 5.3%), seizure (1, 5.3%). Amplitudes > 4 V produced ipsilateral eye lateroversion toward the stimulated side with consequent diplopia (19, 100%). Contralateral CH 2 years post-implant (1, 5.3%), weight loss of 3.5 kg during 3 postoperative months (NS), transient hemiparesis (2, 10.5%)	8.7 years (median)
Torres et al., 2013 [87]	Noticeable sympathetic response in high-frequency stimulation (1, 16.7%), increase in free T4 level 4 months postoperatively (1, 16.7%)	46 months
Piacentino et al., 2014 [66]	Vertigo at 4 V stimulation (4, 100%)	5 years
Benedetti-Isaac et al., 2015 [10]	None	34 months (mean)
Akram et al., 2017 [2]	Transient dizziness (2, 28.6%), nausea (1, 14.3%), intermittent diplopia (1, 14.3%). All were improved with adjustment of the stimulation amplitude. Severe diplopia, oscillopsia, and nystagmus with DBS amplitudes > 2 V (1, 14.3%)	33 months (mean)
Micieli et al., 2017 [56]	Battery site infection (1, 25.0%), transient dystonia in left upper limb improved with changes in stimulation (1, 25.9%), change in intraoperative blood pressure/heart rate (2, 50.0%), transient intraoperative oculomotor movements (1, 25.0%)	27 months (maximum)
Seijo-Fernandez et al., 2018 [75]	Intracerebral electrode breakages (4, 26.7%), systems explantation due a decubitus (2, 13.3%), neck dystonia due to electrode breakage (1, 6.7%)	61.3 months (mean)
Franco et al., 2018 [27]	Stimulation-induced manic symptoms (2, 50.0%), infections (2, 50.0%: 1 associated with skin picking and over connector site, 1 superficial infection over pulse generator). Stimulation of ventral contacts at 130 Hz, 91 μsec, and 3.5 mA or greater induced significant increases in heart rate with no changes in blood pressure, and priapism in the titration phase (1, 25.0%)	6 months
Nowacki et al., 2019 [63]	Stimulation-induced side effects: double-vision, oscillopsia, vertigo, most often double-vision and oscillopsia that was amplitude-dependent and reversible in all patients (6, 100%). Irreversible dysarthria after implantation of the second DBS lead on the right side (1, 16.7%)	91 months (median)
Belvis et al., 2020 [9]	Breakage of 1 electrode (1, 11.1%) and malpositioning of another (1, 11.1%), both needed further surgery	87.4 months
Benedetti-Isaac et al., 2021 [11]	Basal ganglion hemorrhage (1, 5.3%)	> 18 months
Contreras Lopez et al., 2021 [18]	None	12 months (maximum)
Neudorfer et al., 2021 [62]	627 responses to stimulation: tachycardia (194, 30.9%), hypertension (43, 6.9%), flushing (57, 9.1%), sweating, warmth (154, 24.6%), coldness, nausea, phosphenes, and fear	NS
Pastor et al., 2021 [65]	NS	NS
Torres, et al., 2021 [86]	Infection (1, 14.3%), dysfunctional extension cable (1, 14.3%)	144 months (mean)
Escobar Vidarte et al., 2022 [23]	2 (18.2%) patients were unable to tolerate chronic stimulation, infection of the operation site forced explantation in 1 (9.1%), lateral conjugate gaze deviation during the ascent of parameters (1, 9.1%)	4 years (mean)
Venetucci Gouveia et al., 2023 [31]	NS	NS

*CH* chronic headache; *CT* computerized tomography; *DBS* deep brain stimulation; *GA* general anesthesia; *IV* intravenous; *NS* not specified; *V* volt

## Discussion

Reviewing data from 412 patients in 34 studies across ten countries conducted over two decades revealed that the DBS of the hypothalamus is a feasible approach with varying degrees of safety and efficacy for the treatment of a range of medication-refractory conditions, including refractory headache, pathological aggression, mild Alzheimer’s disease, trigeminal neuralgia in multiple sclerosis, Prader-Willi syndrome, and atypical facial pain. In total, 213 patients (51.7%) reported positive outcomes, ranging from 20% to 100% improvements. Overall, 126 patients (30.6%) were successfully treated for headache and 68 patients (16.5%) were treated for aggressive behavior. While it is difficult to find unanimous agreement about the best choices of target based on the diagnosis alone and provide predictive outcomes following these individual targets, it is reassuring to note that DBS has indeed opened new doors for various conditions [5, 7, 90].

Cluster headache is the most severe type of headache affecting up to 0.1% of the population [91]. Chronic and severe headache has been associated with elevated depression, suicidal ideation, and drug addiction [21, 35, 58, 68]. Classically, various approaches, including occipital nerve stimulation, sphenopalatine ganglion stimulation, radiofrequency, cryosurgery, decompression, and neurectomy have been used for the treatment of refractory headaches [1, 3, 38, 41, 51, 52, 57, 83, 88, 92]. Pioneering work by Leone and colleagues to treat CCH by DBS in PH resulted in long-lasting pain relief [39]. While its etiology remains mainly unknown, the peripheral autonomic nervous system, the trigeminovascular system, and the hypothalamus are suggested to be involved in the pathophysiology of CCH [55]. A lack of understanding of the pathophysiology of headache makes understanding the mechanism of DBS in the treatment of these conditions challenging. As CCH is primarily affecting males with a gender ratio of 3-7:1 [24, 53], it was not unexpected that the majority of patients included in the review were males. DBS can modulate a complex network of pain processing areas, creating an antinociceptive effect [55], and future studies can unravel sex-specific dimorphism associated with neural circuits associated with DBS. It is worth emphasizing that the mechanism of pain relief is not due to hormonal changes as investigated in endocrine studies by Schoenen et al [73]. Efficacious stereotactic and functional neurosurgery relies on the accuracy of the device placement procedure. The optimal stimulation sites in DBS for different indications remain to be identified [17, 25].

DBS has been effective in alleviating headache [15, 89]. PH coordinates for the treatment of chronic headache have been classically reported as 2 mm lateral to the midline, 3 mm posterior and 5 mm below the MCP [2]. Akram et al [2] aimed to identify the optimal site for the improvement of headache and they achieved the greatest reduction in headache load at 6 mm lateral, 2 mm posterior, and 1 mm inferior to the MCP, which lays on the trigemino-hypothalamic tract responsible for connecting the trigeminal system and other brainstem nuclei associated with pain modulation and nociception with the hypothalamus, prefrontal, and mesial temporal regions. In contrast, Seijo et al [74] used 4 mm from the third ventricle wall, 2 mm behind the MCP, and 5 mm inferior to the intercommissural line. This area was chosen to avoid damage to the lateral wall of the third ventricle and stimulate several fasciculi that exchange parasympathetic signals between hypothalamus and brainstem. There has been no established targeting strategy in DBS for aggression. Furthermore, discrepancies in the exact demarcation of hypothalamic areas for DBS can be explained by multiple factors, including identification of the target by using surrounding anatomical regions which can introduce heterogeneity and different stimulation amplitude used which can change the area covered around the DBS target.[2, 80] Some studies suggested that efficacious DBS for headache is not restricted to PH and encompasses a widespread area including the midbrain tegmentum, and stimulation may even reach the centromedian thalamus [63]. Anatomical relationship between the hypothalamus and surrounding structures is shown in Figure 2.

**Fig. 3:**
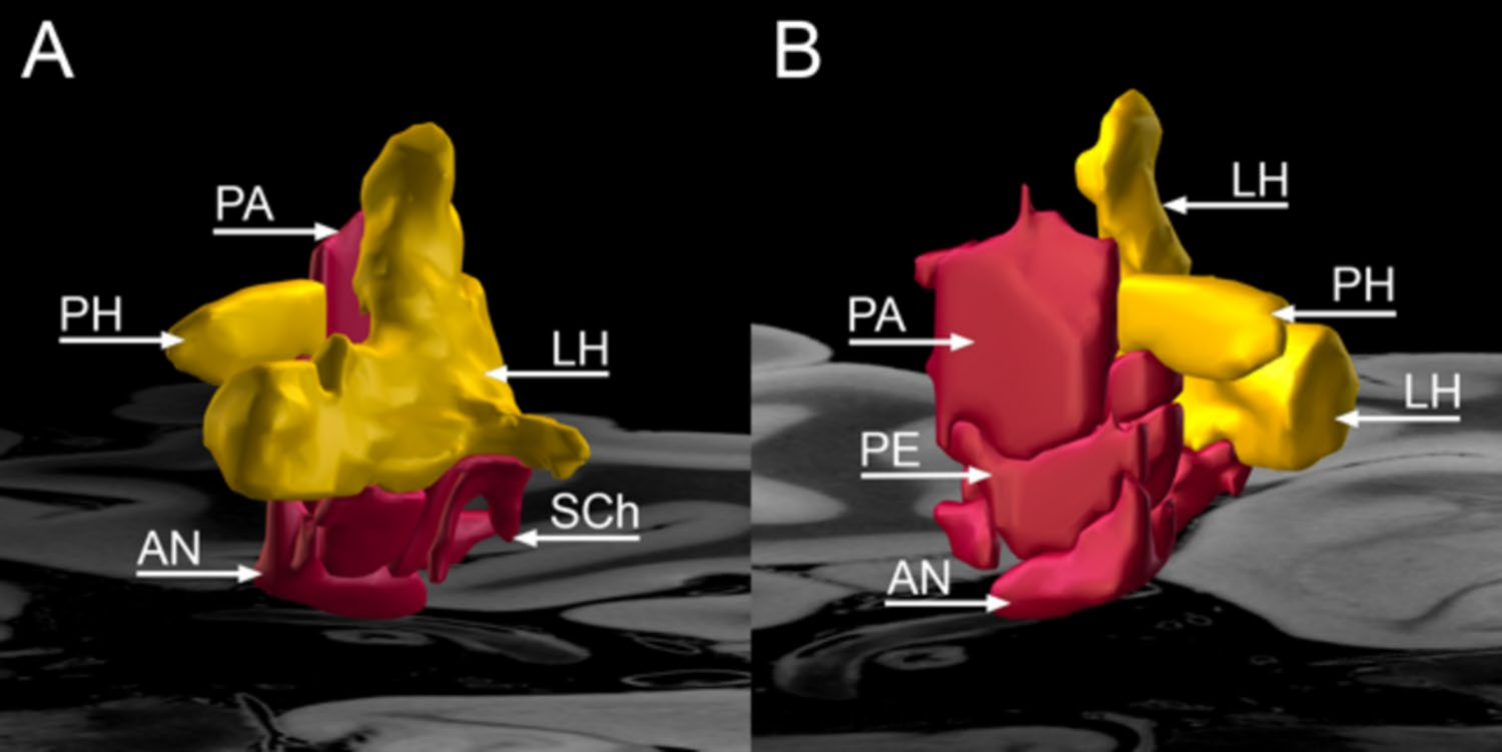
3D reconstruction of hypothalamic nuclei and their anatomical relationships. **A** Oblique posterior view featuring the outer surface of hypothalamic nuclei and **B** medial view featuring the inner surface of hypothalamic nuclei. Hypothalamic nuclei, which have been previously targeted in the literature are featured in yellow. *AN*, arcuate nucleus; *DM*, *LH*, lateral hypothalamus; *PA*, paraventricular nucleus; *PE*, periventricular nucleus; *PH*, posterior hypothalamus; *SCh*, suprachiasmatic nucleus

The anterior boundary of the hypothalamus is defined by the anterior commissure (AC), which is located inferior to the bed nucleus of the stria terminalis (BNST) and the fornix (fx). The latter traverses the hypothalamus and terminates in the mammillary bodies (MM). Along with the mammillothalamic tract (mt), the fornix and mammillary bodies constitute part of the Papez circuit. The lateral boundary of the hypothalamus is formed by the, substantia nigra (SN), subthalamic nucleus (STN), zona incerta (ZI), and red nucleus (RN). The inferior thalamic peduncle (ithp) forms the anterior extension of the lateral boundary. In addition, 3D reconstruction of hypothalamic nuclei and their anatomical relationships is shown in Fig. 3.

Leone, et al [48] reported that bilateral chronic headache was a predictor of poor DBS outcome. Identifying risk factors associated with the failure of hypothalamic DBS can be a leap forward to predicting the patients’ outcomes and identifying the indications of using DBS. In contrast, Leone, et al [46] did not observe differences in efficacy outcome for CH after unilateral or bilateral stimulation.

Medication-resistant aggressive behaviors can have devastating consequences for individuals, their caregivers, and family members [76] It is suggested that aggression occurs due to a reduction in serotonergic transmission in the prefrontal cortex, due to a decreased tolerance to provocative stimuli, resulting in an ineffective inhibitory control of the hyperactivated amygdala [31, 34]. The amygdala prepares the subject for response by signaling periaqueductal gray matter and the hypothalamus for motor activation and hormone synthesis [13, 30]. DBS of the hypothalamus has been used for the treatment of refractory aggressive behavior [10, 11, 18, 23, 28, 56, 65, 86, 87]. The first piece of neurosurgical evidence on the role of the posterior hypothalamus in aggression was reported by Sano and colleagues in 1970 who demonstrated bilateral ablative lesions of PMH in 51 cases with violent and aggressive behavior which was safe and resulted in improvements in 95% of their cases [70]. While the exact neuronal mechanism is not well-understood, it has been proposed that the “triangle of Sano”, comprised of connections between the amygdala, PMH, and the Papez (medial limbic) circuit which enables the communication between the neocortex, limbic structures, and the hypothalamus, is central to the symptom spectrum observed in these patients [16, 23, 32, 84]. More than three decades later, Franzini and colleagues pioneered the implantation of PMH DBS as a non-ablative method for the treatment of aggression [28]. DBS of neural structures for treating aggression is not limited to these structures and other regions, including nucleus accumbens and amygdala have been used as DBS targets for treating aggression [33, 42]. Intraoperative microdialysis during DBS of the posteromedial hypothalamus for refractory intermittent explosive disorder showed that the intraoperative levels of neurotransmitters such as gamma-aminobutyric acid and glycine increased markedly during the stimulation [18].

Escobar Vidarte et al [23] showed that DBS of the PMH can be safe and effective in improving severe and refractory aggressive behavior in patients with long-term intellectual disabilities; symptoms however deteriorated following the depletion of the pulse generator, and battery replacement resulted in recovery. Symptom recurrence with the cease of stimulation due to battery depletion and subsequent improvements in the follow-up after battery replacement also strongly suggests the efficacy of DBS for the treatment of aggression in other cases [23]. Such observations not only demonstrate the direct effect of DBS on treatment but also emphasize the importance of follow-up after the intervention.

Venetucci Gouveia et al [31] demonstrated that the pediatric population had greater improvement in their symptoms compared to the adult population after DBS of PH for aggressive behavior with the optimal target in the more posterior-inferior-lateral region of the PH. Such observations can be due to the higher plasticity of the brain during childhood which makes treating dysfunctional circuitry more feasible and potentially results in long-term beneficial outcomes to restore normal functions; an observation consistent with the findings when treating pediatric dystonia [6, 50, 85]. Future studies including a larger number of pediatric participants can shed more light on utilizing DBS earlier in the life for a potentially better response. Furthermore, differences in efficacy outcomes can be explained by functional and anatomical differences in different patients as well as different etiology behind the aggressive behavior in adults vs pediatric population. Needless to emphasize, though, ethical considerations and multi-disciplinary approach are central when treating this patient group.

Although in the majority of studies patients were suffering from chronic headache, chronic cluster headache, and aggression, three of the studies used this technique for patients with facial pain, trigeminal neuralgia, Obesity associated to Prader-Willi Syndrome and mild Alzheimer’s disease [14, 19, 27, 62]. Broggi et al [14] reported 70% positive outcome for 14 patients with facial pain while Cordella et. Al [19] reported improvement in 100% of 5 patients with trigeminal neuralgia. Franco et al [27] found this method ineffective in treatment of obesity in four patients with Prader-Willi Syndrome. Neudorfer et al [62] did not specify their outcome for patients with mild Alzheimer’s disease. Further studies with larger populations are required to investigate the efficacy of DBS in the hypothalamic region in the treatment of these conditions.

The side effects and complications associated with this surgery were reported mainly as conjugated ocular deviation with extreme verbal response, diplopia, dizziness, and vertigo which happened mainly in high-voltage settings. These complications have been reported in studies with 3 to 144 months of follow up duration. One study also reported death soon after surgery caused by implant-induced hemorrhage [73]. Longer follow ups can identify long-term complications with the DBS of this region. Serious complications such as hemorrhage are rare but can be catastrophic [73]. Technical considerations such as minimizing the use of microelectrode recording when targeting vascular structures as hypothalamus are important to reduce the risks.

## Limitations

Our paper is subject to several limitations. Articles were published in English and were mainly from European centers (73.5%), which does not represent geographical and cultural disparities causing limited access to neurosurgical care [59]. There were several heterogeneities in the papers reviewed, and papers had small number of patients, were not randomized, and outcome assessments were not blinded. Various efficacy outcomes were used to assess the effectiveness of DBS for the same indication. For example, some studies used a 50% threshold in headache attacks [82], whereas others used a 30% reduction [2], causing a heterogeneity of outcomes. In addition, a variety of different assessment tools, such as VAS, OAS, and MOAS were employed to assess the outcome. Different medications used to treat refractory conditions, and various thresholds to assess the responsiveness of patients to medications are additional points which can add to the heterogeneity of results reported. 

## Conclusion

The findings over the past five decades with neuromodulatory approaches have demonstrated that the hypothalamic DBS is feasible with varying degrees of efficacy for a range of disorders including refractory headache and aggressiveness. Larger studies with consistent blinded assessment and randomized design, however, are needed to fully characterize the safety and efficacy of the surgery as well as defining the target population most likely to benefit from it.

## Supplementary Information

Below is the link to the electronic supplementary material.

Supplementary File 1 (DOCX 17.6 KB)

## Data Availability

No datasets were generated or analyzed during the current study.

## Abbreviations

CCH: Chronic cluster headache
CH: Chronic headache
DBS: Deep brain stimulation
HPA: Hypothalamic-pituitary-adrenal axis
MCP: Mid-commissural point
PH: Posterior hypothalamus
PMH: Posteromedial hypothalamus
VAS: Visual analogue scale
