# Supplementary material for: Deep brain stimulation of the hypothalamic region: a systematic review
Source: Acta Neurochir (Wien). 2025 Feb 4;167(1):33. doi: 10.1007/s00701-025-06430-w (PMC11794333; doi:10.1007/s00701-025-06430-w)
Supplement: Supplementary file 1 — (DOCX 17.6 KB) [file 701_2025_6430_MOESM1_ESM.docx]

**Supplementary Table 1.** Search Terms Used for Each Database

| **Database and search results** | **Search terms** |
| --- | --- |
| **PubMed**  1455 | (("hypothalamus"[MeSH Terms] OR "hypothalamus"[All Fields]) AND ("deep brain stimulation"[MeSH Terms] OR ("deep"[All Fields] AND "brain"[All Fields] AND "stimulation"[All Fields]) OR "deep brain stimulation"[All Fields])) OR (("hypothalamically"[All Fields] OR "hypothalamous"[All Fields] OR "hypothalamus"[MeSH Terms] OR "hypothalamus"[All Fields] OR "hypothalamic"[All Fields]) AND ("deep brain stimulation"[MeSH Terms] OR ("deep"[All Fields] AND "brain"[All Fields] AND "stimulation"[All Fields]) OR "deep brain stimulation"[All Fields])) OR (("hypothalamus"[MeSH Terms] OR "hypothalamus"[All Fields]) AND ("deep brain stimulation"[MeSH Terms] OR ("deep"[All Fields] AND "brain"[All Fields] AND "stimulation"[All Fields]) OR "deep brain stimulation"[All Fields])) OR (("preoptic"[All Fields] OR "preoptical"[All Fields]) AND ("deep brain stimulation"[MeSH Terms] OR ("deep"[All Fields] AND "brain"[All Fields] AND "stimulation"[All Fields]) OR "deep brain stimulation"[All Fields])) OR ("Paraventricular"[All Fields] AND ("deep brain stimulation"[MeSH Terms] OR ("deep"[All Fields] AND "brain"[All Fields] AND "stimulation"[All Fields]) OR "deep brain stimulation"[All Fields])) OR ("Suprachiasmatic"[All Fields] AND ("deep brain stimulation"[MeSH Terms] OR ("deep"[All Fields] AND "brain"[All Fields] AND "stimulation"[All Fields]) OR "deep brain stimulation"[All Fields])) OR (("supraoptic"[All Fields] OR "supraoptical"[All Fields]) AND ("deep brain stimulation"[MeSH Terms] OR ("deep"[All Fields] AND "brain"[All Fields] AND "stimulation"[All Fields]) OR "deep brain stimulation"[All Fields])) OR ("Arcuate"[All Fields] AND ("deep brain stimulation"[MeSH Terms] OR ("deep"[All Fields] AND "brain"[All Fields] AND "stimulation"[All Fields]) OR "deep brain stimulation"[All Fields])) OR (("dorsomedial"[All Fields] OR "dorsomedially"[All Fields]) AND ("deep brain stimulation"[MeSH Terms] OR ("deep"[All Fields] AND "brain"[All Fields] AND "stimulation"[All Fields]) OR "deep brain stimulation"[All Fields])) OR (("ventromedial"[All Fields] OR "ventromedially"[All Fields]) AND ("deep brain stimulation"[MeSH Terms] OR ("deep"[All Fields] AND "brain"[All Fields] AND "stimulation"[All Fields]) OR "deep brain stimulation"[All Fields])) OR (("posterior"[All Fields] OR "posteriors"[All Fields]) AND ("deep brain stimulation"[MeSH Terms] OR ("deep"[All Fields] AND "brain"[All Fields] AND "stimulation"[All Fields]) OR "deep brain stimulation"[All Fields])) OR ("Tuberomammillary"[All Fields] AND ("deep brain stimulation"[MeSH Terms] OR ("deep"[All Fields] AND "brain"[All Fields] AND "stimulation"[All Fields]) OR "deep brain stimulation"[All Fields])) OR (("mamillaris"[All Fields] OR "mamillary"[All Fields] OR "mammillary"[All Fields]) AND ("deep brain stimulation"[MeSH Terms] OR ("deep"[All Fields] AND "brain"[All Fields] AND "stimulation"[All Fields]) OR "deep brain stimulation"[All Fields])) |
| **Scopus**  1042 | TITLE-ABS-KEY ( ( hypothalamus AND deep AND brain AND stimulation ) OR ( hypothalamic AND deep AND brain AND stimulation ) OR ( hypothalamus AND dbs ) ) |
| **Web of Science**  651 | ALL=((hypothalamus and deep brain stimulation) OR (hypothalamic and deep brain stimulation) OR (hypothalamus and DBS) ) |
